# Chronic Pain and Stiffness After Total Knee Arthroplasty: A Comprehensive, Phenotype-Based Review of Mechanisms, Diagnosis and Management

**DOI:** 10.3390/jcm15145557

**Published:** 2026-07-15

**Authors:** Furkan Yapıcı

**Affiliations:** Department of Orthopedics and Traumatology, Erzincan Binali Yıldırım University, Erzincan 24100, Turkey; furkanyapici@hotmail.com

**Keywords:** total knee arthroplasty, chronic postsurgical pain, stiffness, arthrofibrosis, range of motion, manipulation under anesthesia, revision knee arthroplasty, neuropathic pain, central sensitization, multidisciplinary pain management

## Abstract

**Background/Objectives:** Total knee arthroplasty (TKA) relieves pain and restores function for most patients with end-stage knee disease; yet, a clinically important minority are left with persistent pain, stiffness or both despite well-fixed, well-aligned implants. These “hidden failures” are poorly captured by implant survivorship but drive disability, dissatisfaction and disproportionate cost—stiff knees incur roughly 1.5–7.5 times the two-year treatment costs of non-stiff knees. This review integrates three usually separate studies within one phenotype-based framework. **Methods:** A structured comprehensive narrative review and thematic synthesis was undertaken; PubMed/MEDLINE, Web of Science and Google Scholar were searched between 30 October 2025 and 1 May 2026, identifying 467 records, of which 239 met the eligibility criteria and informed the synthesis. **Results:** Clinically meaningful chronic pain affects roughly 10–30% of recipients and stiffness approximately 1–7%, with high-impact chronic pain persisting in about one-in-five and neuropathic features in a third to a half of those affected. Contemporary dissatisfaction estimates appear closer to 7–14% than the historical “one-in-five” paradigm, with persistent pain and stiffness the dominant drivers of residual dissatisfaction. Symptoms arise from interacting mechanical, fibrotic, infective, neuropathic, central and psychosocial mechanisms. Six practical phenotypes are proposed—pain-dominant with preserved motion, stiffness-dominant, combined stiff-and-painful, neuropathic-dominant, centrally sensitized, and arthrofibrosis with true mechanical block—and diagnosis, prevention and management are organized around them, spanning risk stratification, surgical technique, manipulation under anesthesia (mean gains ~30–47°, ~6% failure), arthrolysis, revision and multidisciplinary pain care. **Conclusions:** Chronic pain and stiffness are the quiet arithmetic of arthroplasty success—rare on the survivorship curve, common in the clinic. Matching treatment to phenotype and reserving the operating theater for a knee with a mechanical fault to fix rather than a nervous system to calm, offers the clearest path from a technically successful operation to a genuinely successful recovery.

## 1. Introduction

Total knee arthroplasty (TKA) is among the most successful and frequently performed operations in orthopedic surgery, relieving pain and restoring function for most patients with end-stage knee osteoarthritis and other destructive knee conditions. Yet implant survival and radiographic fixation do not fully capture the patient experience: a clinically important minority continue to report persistent pain, stiffness or both despite well-fixed, well-aligned components and technically uncomplicated surgery [1,2,3,4,5]. These “hidden failures” are seldom captured by survivorship curves built on revision or radiographic loosening, but they dominate the patient’s experience, driving disability, dissatisfaction and increased healthcare use [2,4,5,6,7]. This review takes chronic pain and stiffness after TKA—particularly where they coexist in the “stiff, painful” knee—as its central focus, integrating their mechanical, fibrotic, neuropathic, central and psychosocial dimensions within a single phenotype-based framework.

### 1.1. Rationale: Chronic Pain and Stiffness as “Hidden Failures”

Catastrophic complications such as periprosthetic joint infection (PJI) and gross instability are relatively uncommon; chronic pain and stiffness are far more frequent and more insidious. Clinically meaningful stiffness affects roughly 1–7% of TKAs and, in its severe early forms, carries worse survivorship and triggers cascades of physiotherapy, manipulation under anesthesia (MUA), arthrolysis or revision [8,9,10,11,12,13]; clinically meaningful persistent pain affects some 10–30% of recipients, with a larger fraction reporting milder symptoms [1,2,14,15,16]. Detailed epidemiology, dissatisfaction data and economic burden are developed in Section 4.

Even when uncommon in absolute terms, these problems are consequential. Fixed or limited flexion increases quadriceps demand, alters patellofemoral loading and impairs basic tasks such as rising from a chair or climbing stairs [8,10,11]; patients with high preoperative pain and psychological distress consume more rehabilitation, undergo MUA more often, and a sizeable minority fail to achieve even moderate functional improvement by six months [2,17]. Pain and stiffness are tightly interwoven rather than independent: revision series for stiffness almost uniformly report that patients present with both, that pain relief—not motion gain—is the usual motivation for surgery, and that this “stiff, painful” group carries the highest risk of repeated reoperation and ultimate failure [3,9,18,19,20].

From the patient’s standpoint, dissatisfaction maps poorly onto any single parameter such as range of motion (ROM) or radiographic alignment [4,5]. Arthrofibrosis represents one pole—a biologically aggressive process of periarticular fibrosis, capsular contracture and adhesion in which pain and motion loss are tightly coupled; isolated patellofemoral or neuropathic pain with near-normal ROM lies at the opposite pole, and a non-trivial fraction of painful knees defy mechanical explanation, implicating neuropathic and centrally mediated mechanisms [1,21,22,23]. Between these poles pain and stiffness reinforce one another, so that high preoperative pain, anxiety and depression predispose to both [2].

### 1.2. Scope, Aims and Key Questions

Historically, TKA success was defined almost exclusively by implant survival and radiographic stability [18,21]. As implants improved, validated pain and function instruments revealed that radiographic success did not equate to satisfaction, and that expectations, baseline function, mental health and comorbidity independently predicted outcome [4,5,17]; in parallel, the “stiff TKA” evolved from a purely technical complication into a multifactorial problem spanning preoperative limitation, gap imbalance, component malposition, periarticular fibrosis and suboptimal rehabilitation [8,11,24,25]. Chronic pain and stiffness sit at the center of this shift, frequently being why a technically “successful” arthroplasty is experienced as a failure.

Against this background, this review adopts a broad biopsychosocial perspective rather than a narrow focus on arthrofibrosis or on chronic pain alone. It integrates mechanical and structural factors, fibrotic biology, peripheral nociceptive, neuropathic and central mechanisms, psychosocial and rehabilitation determinants, surgical prevention and treatment, and patient-centered and health-system consequences [1,2,3,4,5,8,9,10,11,17,18,19,20,21,22,24,25,26,27,28,29,30,31]. Within this framework it addresses seven questions: how chronic pain, stiffness and arthrofibrosis should be defined and distinguished, including “pain-limited” versus “true mechanical” stiffness; their epidemiology and burden, and their contribution to residual dissatisfaction, including the historical “one-in-five” paradigm and contemporary estimates closer to 7–14% depending on definitions; the patient-, disease- and surgery-related risk factors that drive them; whether clinically meaningful phenotypes of the painful and/or stiff TKA can be defined; which diagnostic pathways best separate mechanical from biological and psychosocial causes; what evidence supports prevention and treatment across the perioperative pathway, including the role and limits of revision when components are sound but symptoms persist; and where the main knowledge gaps and research priorities lie.

The central premise is that chronic pain and stiffness after TKA should be approached not as a single complication or a binary distinction between “failed implant” and “unexplained pain”, but as overlapping clinical phenotypes along a continuum (detailed in Section 7). Accordingly, this work is structured as a comprehensive thematic synthesis integrating epidemiology, mechanisms, risk factors, diagnosis, prevention, treatment and research priorities into a clinically usable roadmap for reducing the burden of chronic pain and stiffness after TKA.

## 2. Methods: Structured Comprehensive Narrative Review and Thematic Synthesis

### 2.1. Design and Objectives

This article was designed as a structured, comprehensive narrative review and thematic synthesis rather than a formal systematic review or meta-analysis. Its objective was to integrate three studies usually considered separately—chronic postsurgical pain (CPSP) after TKA, postoperative stiffness and arthrofibrosis, and dissatisfaction despite technically successful arthroplasty—within a single biopsychosocial, phenotype-based framework emphasizing clinically actionable synthesis rather than quantitative pooling. A formal meta-analysis was not planned because definitions, populations, interventions, follow-up and outcome measures vary substantially across this evidence base [3,10,12,13,14,18,19,23,29,32,33,34,35,36]. These aims were organized into five linked domains: definitions; epidemiology, burden and overlap; mechanisms; phenotypes; and phenotype-aligned prevention, diagnosis and management.

### 2.2. Literature Sources and Search Strategy

Searches were conducted in PubMed/MEDLINE, Web of Science and Google Scholar between 30 October 2025 and 1 May 2026, restricted to full-text English-language articles, with no lower date limit so that early landmark stiffness and revision series could be included alongside recent systematic reviews, randomized trials and mechanistic studies. The strategy combined procedure terms (total knee arthroplasty/replacement, revision) with pain, stiffness/arthrofibrosis and determinant, diagnostic and outcome terms, and was refined iteratively, using existing systematic reviews as both evidence sources and anchors for backward and forward citation tracking and manual reference-list screening [13,14,23,32,33,34,35,36,37,38,39]. Gray literature was not formally searched, and conference abstracts were considered only when linked to subsequent full-text publications. Landmark surgical series on the stiff TKA, revision for stiffness, MUA and arthrolysis were specifically traced through citation networks, as they remain central to current algorithms despite predating contemporary CPSP terminology [3,10,12,18,19,27,29].

### 2.3. Eligibility and Evidence Domains

**Figure 1:**
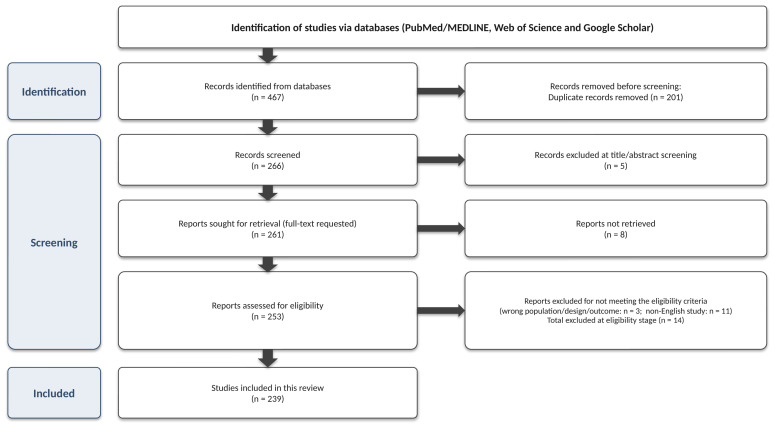
Flow diagram of study identification, screening and inclusion.

Studies were eligible if they involved adults undergoing primary or revision TKA and addressed at least one domain: CPSP (pain persisting or newly emerging ≥ 3 months after surgery); postoperative stiffness or arthrofibrosis (defined by ROM thresholds, flexion contracture, or need for MUA, arthrolysis or revision); combined pain-and-stiffness outcomes; diagnostic evaluation; prevention and treatment; or patient-reported outcomes, satisfaction and economic burden. Eligible designs ranged from randomized and quasi-experimental trials through prospective and retrospective cohorts, case–control, cross-sectional and clinically relevant case series to systematic reviews, meta-analyses, guidelines, consensus statements and health-economic evaluations; mechanistic and translational studies were included when they directly informed the biology of arthrofibrosis, central sensitization or neuropathic pain. Studies confined to unicompartmental or patellofemoral arthroplasty, to acute pain without follow-up, or to purely radiographic or biomechanical endpoints were excluded. From 467 records identified, 239 studies met eligibility after duplicate removal and screening; the selection process is summarized in Figure 1. In addition to these 239 included studies, a small number of methodological and definitional sources are cited, giving a total of 241 references.

### 2.4. Data Charting and Thematic Synthesis

Included articles were charted using a structured framework capturing bibliographic details, design, setting, sample size, primary versus revision status, definitions of pain/stiffness/arthrofibrosis, follow-up, interventions, outcome measures, key findings and limitations. Studies were first grouped as pain-focused, stiffness-focused or mixed—an organizing device rather than a claim of separability—then mapped onto cross-cutting mechanistic categories (mechanical–structural, fibrotic, infective, neuropathic, central/nociplastic and psychosocial). Thematic synthesis then compared definitions and epidemiologic estimates, organized mechanistic findings into these domains, developed clinical phenotypes by integrating ROM patterns, pain quality, mechanical findings and psychosocial features, and aligned diagnostic and management strategies with these phenotypes to generate practical algorithms.

### 2.5. Evidence Weighting and Limitations of the Design

Because this was a structured comprehensive narrative review, no standardized risk-of-bias tool was applied and no quantitative meta-analysis was performed. Evidence was weighted pragmatically: systematic reviews, meta-analyses, randomized trials and large prospective cohorts were prioritized for prevalence, risk factors and intervention effects; registries and large retrospective cohorts described real-world incidence, reoperation burden, dissatisfaction and cost; and smaller series, narrative reviews and expert opinion informed phenotype construction and practical algorithms where higher-level evidence was lacking. Several limitations follow: the search was broad and structured but not preregistered, and selection was not by dual independent screening, so publication, language and selection biases cannot be excluded; and the literature is highly heterogeneous, with inconsistent thresholds for chronic pain, stiffness and arthrofibrosis and variable follow-up, which limits direct comparison and weakens prediction models. The algorithms proposed here should therefore be read as pragmatic, evidence-informed frameworks rather than formal guidelines, requiring prospective validation with standardized definitions, core outcome sets and patient-centered endpoints.

## 3. Definitions and Conceptual Framework

Neither “chronic pain” nor “stiffness” after TKA has a single, universally accepted definition: authors apply different time thresholds for chronic pain, different ROM cut-offs for stiffness, and varied histopathological or imaging criteria for arthrofibrosis [8,9,12,13,32,34,35], while modern work frames chronic postsurgical pain (CPSP) as a multidimensional biopsychosocial construct rather than a simple pain score [14,23,40,41,42]. These inconsistencies complicate prevalence estimates, comparison between studies and interpretation of treatment outcomes. This section sets out the working definitions used throughout the review (summarized in Table 1) and embeds them in the biopsychosocial framework that organizes the sections that follow.

### 3.1. Typical Recovery and the Threshold for Chronicity

TKA reliably improves pain, function and health-related quality of life for most patients. In uncomplicated recovery, the greatest gains in pain and basic function occur within the first three months, followed by slower improvement to about one year and relative plateau thereafter [4,5,41,43]. ROM follows a similar course: flexion often declines transiently in the early postoperative period, then recovers over three to six months, with many patients achieving a functional arc of roughly 100–120° and minimal residual flexion contracture [9,10,34]. Recovery speed varies with age, comorbidity, preoperative function and acute postoperative pain, and most patients regain basic transfers, assisted ambulation and stair negotiation within the first one to two weeks [44,45]. In this review, typical recovery denotes a trajectory in which pain decreases substantially by three months, ROM progresses toward at least approximately 100–110° with less than 10–15° of flexion contracture, essential activities of daily living are regained, and no major complication such as infection, fracture, gross instability or early loosening is present. Persistent pain, persistent ROM limitation, or both beyond this expected window form the focus of the review.

### 3.2. CPSP, Stiffness and Arthrofibrosis: Definitions and Functional Thresholds

Following the International Association for the Study of Pain, CPSP is defined as pain that develops or worsens after surgery, persists for at least three months, and is not better explained by infection, malignancy or a pre-existing pain condition in the same region [46]; the three-month threshold is appropriate for knee arthroplasty because most pain improvement occurs earlier [41]. TKA-specific studies nonetheless use heterogeneous operational definitions, with some applying six- or twelve-month thresholds and varied intensity cut-offs such as visual analog or numerical rating scale ≥ 3/10 or ≥30/100 [14,36,40,41]. Contemporary reviews consistently place clinically meaningful persistent pain at approximately 10–30%, with milder symptoms in a larger fraction [14,15,16,40,41,42,47]. This review treats CPSP as a clinical syndrome requiring phenotyping rather than a diagnosis of exclusion, since persistent pain is often driven by identifiable mechanical, nociceptive, neuropathic, central or psychosocial factors [1,2,14,16,22,23,36,40,41,42,48,49,50,51]. Stiffness, by contrast, is a clinical description rather than a disease, defined variously by absolute ROM, flexion contracture, failure to reach functional milestones, or the need for MUA or arthrolysis [8,9,12,13,32,33,34]; here it is used as an umbrella term for restricted motion that compromises function, typically flexion < 90–100° and/or fixed flexion contracture > 10–15°. Definitions vary widely across the literature: older surgical series often used severe cut-offs such as flexion < 70–80° or total arc < 90°; whereas, more recent studies use flexion < 90–100°, flexion contracture > 10–15°, or the need for MUA, arthrolysis or revision [9,10,12,13,34]. Arthrofibrosis denotes a more specific subset: the international consensus defines postoperative knee fibrosis as limited flexion and/or extension caused by new intra-articular scar tissue not present at surgery [38], and it is best conceptualized as a triad of pain, motion loss and functional deficit that does not resolve with normal rehabilitation, reflecting a biologically distinct, excessive fibrotic response rather than ordinary scarring [52]. It should be distinguished from stiffness caused primarily by component malposition, instability, infection, heterotopic ossification or extra-articular deformity, which require different management. AIS is therefore a clinical label applied after exclusion of recognized causes; whereas, arthrofibrosis denotes a fibrotic biological substrate that may underlie some, but not all, AIS cases [38,52]. Because the functional cost of any given arc depends on the task—roughly 65° for level walking, 90° for many basic activities of daily living, 100–110° for comfortable stair climbing and chair rise, and more than 120° for kneeling, squatting or some cultural and religious activities [10,53,54]—even modest ROM differences (for example 80° versus 100°) can substantially affect participation. Working definitions, severity bands and these thresholds are collated in Table 1.

### 3.3. Pain-Limited Versus True Mechanical Stiffness

A clinically crucial distinction is whether reduced motion is fixed or pain-mediated, because the two appear similar on cursory examination but demand different treatment. In pain-limited stiffness, clinic ROM is markedly reduced but the passive arc is substantially greater under relaxation or anesthesia, the end-feel is soft or inconsistent, and motion improves with optimized analgesia, reassurance, graded exposure, intensive physiotherapy, bracing or neuromuscular stimulation, without surgery [27,28,41,42]. This pattern is particularly relevant where central sensitization, catastrophizing, fear of movement or neuropathic pain drives protective under-use. Bhave and colleagues described complex post-arthroplasty patients with flexion deficits, fixed flexion, quadriceps inhibition and nerve-related pain who improved substantially with individualized rehabilitation, bracing and neuromuscular intervention rather than major revision, underscoring that much early motion loss is functionally recoverable [28]. True mechanical stiffness or arthrofibrosis, by contrast, shows a restricted passive arc both awake and anesthetized, a firm or abrupt end-feel, and imaging or intra-operative evidence of capsular contracture, adhesions or heterotopic ossification; depending on maturity it is addressed with carefully selected manipulation or with arthrolysis or revision, as detailed in Section 8.3 and Section 10 [10,29,35,52]. The two are separated in practice by comparing active with passive ROM (awake and, when needed, under anesthesia), assessing end-feel, gauging the response to short-term intensive therapy, and evaluating pain behavior and kinesiophobia. Equally important, pain and stiffness do not coincide in every knee: some patients have near-normal ROM yet disabling pain from malrotation, patellofemoral overload, low-grade infection, or neuropathic and central mechanisms, while others have a genuinely stiff, painful knee in which pain occurs at the extremes of motion and arthrofibrosis, malposition with gap imbalance, heterotopic ossification or extra-articular contracture predominate [1,9,10,18,21,22,23,35]. Section 7 develops these patterns into a more granular phenotypic classification.

### 3.4. A Biopsychosocial Model and the Pain–Stiffness Overlap

Risk-factor studies, mechanistic experiments and longitudinal cohorts together support a biopsychosocial model in which chronic pain and stiffness after TKA emerge from three interacting domains [2,4,5,14,23,35,52]. The first is pain mechanism, encompassing a local nociceptive component (synovitis, polyethylene wear, loosening, patellofemoral overload, occult infection), a neuropathic component from injury to periarticular nerves such as the infrapatellar branch of the saphenous nerve, and a central component of sensitization and impaired descending modulation; the relative weight of each varies across phenotypes and over time, with susceptible patients transitioning from joint-centric to more widespread, centrally facilitated pain (Section 5) [1,22,23,35,40,48,50,51]. The second is fibrosis biology: arthrofibrosis is an exaggerated, partly immune-mediated wound-healing response involving myofibroblast activity, dysregulated TGF-β signaling and excess collagen deposition, promoted by bleeding, synovitis, infection, repeated trauma or gap mismatch and by systemic predispositions such as prior arthrofibrosis, inflammatory disease, diabetes and probable genetic susceptibility (Section 5) [35,38,52]. The third is psychosocial context: preoperative expectations, mental health and comorbidity predict pain and function, while pain catastrophizing, anxiety, depression, kinesiophobia and maladaptive sleep–pain behavior predict CPSP and the need for MUA [2,4,5,14,41,42,55]. These domains are not additive but mutually reinforcing: revision series for stiffness almost uniformly report concurrent pain, with pain relief—not motion gain—the usual driver of satisfaction [3,10,18,19,35], and the pain–stiffness loop detailed in Section 5 means CPSP and arthrofibrosis frequently coexist and aggravate one another [2,8,10,11,42]. Arthrofibrosis is therefore best regarded as both a mechanical and a pain disorder requiring integrated management, and CPSP cannot be interpreted without first establishing whether a knee is mechanically normal, stiff, unstable, inflamed or infected [23,35,52]. This overlap structures the remainder of the review: Section 4, Section 5 and Section 6 address epidemiology, mechanisms and risk factors, Section 7 defines clinical phenotypes, and Section 8, Section 9, Section 10, Section 11, Section 12 and Section 13 present diagnostic, preventive and treatment pathways that disentangle, yet pragmatically manage, the intertwined drivers of chronic pain and stiffness after TKA.

## 4. Epidemiology, Clinical Burden and Health Economics

Chronic pain and stiffness after TKA lie at the intersection of three large public-health problems: the high and rising volume of arthroplasty (already >700,000 primary TKAs annually in the United States and projected to approach 0.94–1.26 million by 2030), the high background prevalence of musculoskeletal pain, and the growing expectations of an aging but active population [6,56,57]. At this scale, even modest percentages translate into hundreds of thousands of affected patients each year (Table 2). Four messages recur: chronic pain of at least moderate intensity affects roughly 10–30% of recipients; stiffness and arthrofibrosis are less common (~1–7%) but disproportionately drive manipulation, arthrolysis, revision and readmission; the two coexist far more often than not, particularly in revision and arthrolysis series where the stiff knee is rarely painless; and together they are major contributors to dissatisfaction and “hidden failure” even when implants are radiographically stable [13,14,15,52,58,59].

### 4.1. Prevalence of Chronic Pain After TKA

Early single-surgeon cohorts already hinted at a troublesome minority, with significant pain in ~13% of Brander’s and ~7% of Ritter’s otherwise satisfactory knees once obvious causes were excluded [1,2]. Contemporary work confirms a substantial signal: cross-sectional and systematic-review data report some persistent pain in a large fraction and moderate-to-severe long-term pain in a clinically important minority, varying by definition and follow-up—around one fifth of patients at three months and roughly 12–15% at two years [15,16,60]. Programs around the STAR trial and other cohorts cluster moderate-to-severe chronic pain at 10–34% (recent studies 15–29%), with about one fifth meeting IASP-based CPSP criteria at 3–6 months [41,42,58,61]. In aggregate, any persistent pain affects 30–50% of patients in some cohorts, moderate-to-severe CPSP 10–30%, and neuropathic features one third to one half of those affected [15,16,42,51,58]; a large 2026 longitudinal cohort found high-impact chronic pain persisting in about one in five, with preoperative high-impact pain the strongest predictor [62].

### 4.2. Prevalence of Stiffness and Arthrofibrosis

Reported stiffness prevalence depends heavily on definition, ranging from 1% to >10% across inconsistent criteria [52], with cohort estimates spanning ~1.3–6.1% [12,59,63] and more stringent final-follow-up definitions (e.g., flexion contracture ≥ 15° and/or flexion < 75°) yielding ~1–2% [9]. Early catastrophic stiffness is rare: in the Mayo experience ~0.3% of 12,850 TKAs had an arc < 50° at 2–4 weeks and ~0.1% progressed to fibrous ankylosis, both with poor outcomes [10]. For narrowly defined acquired idiopathic stiffness (AIS), Tibbo’s systematic review (35 studies, 48,873 TKAs) reported a 4% prevalence, higher in women and BMI ≥ 30 kg/m^2^, with stiffness accounting for up to 58% of non-revision reoperations and over 25% of 90-day readmissions [13]. Arthrofibrosis is a narrower pathobiological construct distinguished by international consensus from stiffness due to malposition, instability or extra-articular pathology, so its true incidence is uncertain [38,52]. The defensible summary is that functionally defined stiffness affects roughly 1–7% of patients, AIS about 4%, and catastrophic early stiffness ≤ 0.3%—small fractions representing large absolute numbers and a disproportionate share of reinterventions.

### 4.3. Co-Occurrence and the “Hidden Failure” Group

Stiffness and pain are interwoven rather than independent: revision and arthrolysis series consistently show that the stiff knee is rarely painless and that pain relief, more than motion gain, drives satisfaction—stiff knees are revised for combined pain and disability, flexion may rise substantially after arthrolysis (e.g., 66° to 107°) yet often remains limited, and stiffness is itself defined as a painful limitation of motion [9,10,12,18]. At population level these patients form the “hidden failure” group—radiographically sound, unrevised, yet limited and dissatisfied: 20–30% fail to achieve even moderate functional improvement despite no mechanical failure [4,5], and registries record 17–19% of unrevised knees rated unsatisfied or uncertain, tracking insufficient pain relief and limited function rather than radiographs [64,65]. Stiffness is also a leading driver of reintervention—up to 58% of non-revision reoperations and >25% of readmissions in some series [13], with revision the commonest reoperation (7.6%) in Olsen’s stiff cohort—confirming that stiff-and-painful knees consume a disproportionate share of surgical and rehabilitation resources [59].

### 4.4. Impact on Function, HRQoL and Satisfaction

Because comfortable walking, stair use and chair transfers require roughly 90–100° of flexion and higher-demand tasks more than 120° (Table 1), patients with flexion < 90° or fixed flexion contracture > 15–20° face marked limitation, abnormal gait and increased energy expenditure [10,52]. At population level TKA reliably improves health-related quality of life (HRQoL)—in a UK cohort of >120,000 knees, mean Oxford Knee Score rose from ~19 to 35 and EQ-5D from 0.414 to 0.734 [66]—but these means conceal a long tail: a substantial minority remain dissatisfied or cannot resume regular activity, and high-pain patients hold far worse utility scores (17% in health states valued as worse than death versus <1% of low-pain patients) [61,66,67,68]. Even in young, active recipients, 91% were satisfied with pain relief yet only 66% felt their knee was “normal” [68]. Pain and stiffness compound one another, with dissatisfied patients consistently showing worse pain, function, ROM and stiffness [6] and recipients remaining more limited than age-matched controls in kneeling, squatting and stair descent [67].

These threads converge on the much-cited “20% dissatisfied” paradigm. The Swedish register (17% of 25,275 knees) [64], the Ontario cohort (19%) [65], with supporting registry, risk-factor and outcome data [69,70,71,72,73,74,75,76,77], and Gunaratne’s systematic review (“one dissatisfied patient for every five TKAs”) [78] established the figure, but DeFrance and Scuderi’s contemporary systematic review of 21 studies and 25,235 patients estimated an average dissatisfaction rate of 10%, falling to 7.3% when major complications were excluded and remaining 13–14% even when neutral responses were counted as dissatisfied [6]. The true contemporary proportion is therefore probably closer to 7–14% than 20%, yet the profile of dissatisfaction is unchanged—driven by inadequate pain relief, residual stiffness and unmet expectations (with reported risk ratios up to 10.8 for unmet expectations) and by psychological factors such as catastrophizing, anxiety and depression [6,7,42,79]. Standard PROMs capture average benefit but show ceiling effects, mismatch with satisfaction and limited coverage of neuropathic, psychosocial and stiffness-specific domains, arguing for complementary measures [7,67,68,79].

### 4.5. Economic Burden

The direct costs of stiffness arise from added procedures, imaging, injections and prolonged rehabilitation. In Olsen’s analysis, stiff patients used substantially more physiotherapy, medication, bracing and clinic visits and, over the two years after surgery, incurred costs 1.5–7.5 times those of non-stiff patients, with dual-component revision markedly more expensive in stiff knees ($65,771 versus $48,287) [59]. Fewer data quantify chronic-pain care, but patients are described “cycling” through repeated consultations, long-term analgesics, imaging, injections, nerve blocks, radiofrequency procedures and occasional neuromodulation [61,80,81]. Against this inefficient usual care, structured pathways can be cost-effective: over four years the STAR pain pathway achieved a higher rate of acceptable symptom state at lower mean NHS cost, with an incremental net monetary benefit of ~£3525, with mean NHS costs ~£1001 lower than usual care, and a ~94% probability of cost-effectiveness at a £20,000/QALY threshold [58,82]. Indirect costs—lost productivity, early retirement, disability claims and caregiver burden—are rarely quantified but clearly substantial [61,83,84]. Overall, chronic pain and stiffness after TKA are not rare complications but a sizeable minority of outcomes consuming a disproportionate share of healthcare use and cost, making their prevention and management central to patient-centered evaluation of TKA success.

## 5. Mechanisms: Mechanical, Fibrotic, Neuropathic, Central and Psychosocial Drivers

Chronic pain and stiffness after TKA arise from interacting mechanical, fibrotic, nociceptive, neuropathic, central and psychosocial mechanisms rather than a single pathway, and although often discussed separately they are tightly coupled in practice: component malrotation, gap imbalance, patellofemoral overload or instability generate abnormal stresses, synovitis and nociceptive pain; in susceptible patients the same triggers promote a profibrotic wound-healing response, capsular contracture and loss of range of motion (ROM); and persistent pain then drives guarding and deconditioning while stiffness raises joint reaction forces and amplifies pain [8,10,22,24,35,52].

This section is mechanistic rather than chronological. Mechanical and fibrotic mechanisms explain many stiffness-dominant and stiff-painful presentations; peripheral neuropathic and central mechanisms explain many pain-dominant or disproportionate pain presentations; and psychosocial and behavioral factors influence whether local tissue pathology resolves, stabilizes or evolves into chronic disability. These domains are not mutually exclusive. Most problematic TKAs occupy several domains simultaneously, and the dominant driver may change over time.

### 5.1. Mechanical and Structural Contributors

Mechanical factors remain fundamental in the pathogenesis of both persistent pain and stiffness after TKA. They may produce symptoms directly through abnormal loading, impingement, instability, patellofemoral overload or mechanical block, and indirectly by triggering synovitis, bleeding, pain-related guarding and fibrotic scarring. Importantly, clinically relevant mechanical problems may be subtle: a knee can appear acceptably aligned on plain radiographs while still having clinically important rotational error, flexion-space overstuffing, joint-line elevation, patellofemoral maltracking or mid-flexion instability [11,22,24,26].

Rotational alignment is particularly important in the painful TKA. Barrack et al. found that combined internal rotation of the femoral and tibial components was strongly associated with anterior knee pain; whereas, coronal alignment, isolated femoral rotation and static patellar tilt were less discriminating [22]. Internal rotation alters the functional trochlear groove, raises lateral patellofemoral contact pressure and tightens the lateral retinaculum, provoking pain during stair descent, chair rise and loaded flexion—presenting as pain-dominant anterior knee pain with preserved ROM, or as pain-limited stiffness in a patient who guards against painful flexion.

Flexion–extension gap imbalance is a central mechanism in stiff-and-painful TKAs: a tight extension gap predisposes to flexion contracture; whereas, a tight flexion gap limits flexion and produces pain at terminal bending [8,11,12,24]. Asymmetric gaps may generate instability or paradoxical motion, experienced as pain, weakness, apprehension or giving way. Joint-line elevation disrupts collateral isometry, reduces quadriceps efficiency and may contribute to mid-flexion instability and anterior knee pain [11,24,85], while tibial slope errors alter flexion-space mechanics—reduced slope tightening the flexion gap, excessive slope contributing to flexion instability, particularly in cruciate-retaining designs [8,24,86].

Component sizing and sagittal positioning also matter. Oversizing of the femoral component, anterior overhang, excessive polyethylene thickness or flexion-space overstuffing can create a hard end point in flexion and increase quadriceps demand. Lo et al.’s report of severe stiffness due to an oversized femoral component illustrates how a localized technical issue can produce marked flexion limitation resistant to rehabilitation until the mechanical problem is corrected [26]. These observations support an important principle: stiffness is not always a biological scar problem. It may be a mechanical consequence of a tight, overstuffed or malbalanced arthroplasty.

The patellofemoral joint is a frequent source of persistent pain and a key link between mechanical error and functional limitation. Overload may result from component internal rotation, anterior overstuffing, excessive patellar thickness, maltracking, patella baja, lateral retinacular tightness or unaddressed patellar disease, and even small increases in patellofemoral thickness raise contact forces and quadriceps demand during flexion [21,22,85,87,88]. Boyd et al. found that unresurfaced patellae were associated with chronic peripatellar pain and secondary resurfacing despite otherwise acceptable alignment and motion [21]. Local patellofemoral pain can therefore drive dissatisfaction independently of global stiffness, while pain with loaded flexion may promote avoidance, quadriceps inhibition and secondary capsular shortening.

Instability, impingement, heterotopic ossification and extra-articular constraints further complicate the mechanical landscape. Coronal, sagittal or mid-flexion instability can cause recurrent effusion, synovitis, apprehension, quadriceps inhibition and pain [11,24,35], and mechanical impingement from retained osteophytes, cement fragments, posterior capsular scarring, patellar clunk or soft-tissue entrapment produces sharp pain at reproducible angles. Heterotopic ossification is common radiographically but clinically important mainly when extensive, when it may tether the extensor mechanism or block flexion or extension [89,90]. Extra-articular causes—prior fractures, osteotomies, extensor-mechanism scarring, hamstring or hip-flexion contracture, lumbar disease and thigh fascial adhesions—may limit apparent knee motion despite an acceptable implant [91,92], and in complex stiff TKAs intra-articular arthrofibrosis and extra-articular contracture frequently coexist.

### 5.2. Arthrofibrosis Biology and Mechanical–Fibrotic Feedback

Arthrofibrosis is the principal biological mechanism linking surgery, inflammation, pain and persistent stiffness. It should not be understood simply as “too much scar” or as the extreme end of normal healing. The international consensus on knee fibrosis defines postoperative fibrosis as limited ROM in flexion and/or extension caused by new intra-articular scar tissue not present at the index procedure [38]. In the TKA setting, arthrofibrosis is characterized by excessive scar formation, capsular contracture, adhesions, myofibroblast activity and extracellular matrix deposition, leading to persistent, function-limiting ROM loss [35,38,52].

Histopathologic and molecular studies support arthrofibrosis as a true fibrotic disorder. Revision specimens show dense collagenous tissue replacing synovium and capsule, α-smooth-muscle-actin-positive myofibroblasts, fibrocartilaginous metaplasia and sometimes heterotopic bone [29,52,93]. Bayram et al. used RNA sequencing to identify a distinct molecular profile, with up-regulation of extracellular-matrix remodeling, inflammatory chemokine, angiogenesis and profibrotic pathways [94], and reactive oxygen and nitrogen species may further amplify fibroblast activation [95,96].

Immune-cell data add another layer. Immunohistochemistry shows increased T lymphocytes, macrophages and mast cells in arthrofibrotic tissue, and Limberg et al. found that immune-cell populations differ from aseptic-loosening tissue [93,97,98], with adaptive Th17-type and B-cell responses also implicated [99]. These findings support a view of arthrofibrosis as a chronic immune-mediated fibrotic disorder, with dysregulated transforming growth factor-β signaling, persistent myofibroblast activation and failed resolution of wound healing [100]. Conversely, synovial fluid may lack the leukocytosis or biomarkers of infection or inflammatory arthritis, suggesting many cases are primarily fibrotic rather than overtly inflammatory [101].

Inflammatory and infective triggers nevertheless remain important. Hemarthrosis, persistent synovitis, wound problems, repeated manipulations, multiple prior surgeries and low-grade periprosthetic joint infection (PJI) can all increase cytokine activity and tissue trauma, creating a milieu favorable to fibroblast activation [35,52]. Low-grade PJI deserves particular attention because it may mimic idiopathic arthrofibrosis. Occult infection and unexpected positive cultures have been reported in some knees revised for presumed aseptic stiffness or arthrofibrosis [102,103]. Biofilm-related low-grade inflammation may promote fibrin deposition, synovitis and profibrotic cytokine release, blurring the boundary between infection and fibrosis. The clinical implication is clear: infection must be excluded before stiffness is labeled idiopathic arthrofibrosis.

Mechanical and fibrotic mechanisms interact bidirectionally. Malrotation, gap imbalance, patellofemoral overload or instability may cause abnormal loading and synovitis, creating a biological stimulus for fibrosis. Once fibrosis develops, capsular contracture and adhesions restrict motion, increase joint stresses and generate pain. Pain encourages guarding and under-use, which reduces tissue excursion and permits maturation of adhesions [8,10,22,24,35,52]. This feedback loop explains why some stiff TKAs remain refractory after a single intervention: mature arthrofibrosis may persist after correction of a mechanical trigger, while arthrolysis alone may fail if malrotation, overstuffing or instability remains uncorrected. The mechanism is often not mechanical versus biological, but mechanical stress plus abnormal biological healing in a susceptible host.

### 5.3. Peripheral Nociceptive and Neuropathic Pain Mechanisms

Persistent pain after TKA may arise from ongoing nociceptive input, peripheral neuropathic mechanisms or both. Nociceptive pain reflects activation of peripheral nociceptors in synovium, bone, capsule, tendons, ligaments and periarticular soft tissues. Neuropathic pain reflects injury or dysfunction of peripheral nerves, neuroma formation or complex regional pain syndrome (CRPS)-like mechanisms. These processes often coexist with mechanical and fibrotic pathology, but they may also dominate in patients with preserved ROM and apparently acceptable implants [16,23,50,51,104,105,106,107,108,109].

Even in radiographically stable TKAs, nociceptive input may persist from synovitis, effusion, fat-pad irritation, tendinopathy, patellofemoral overload, instability, subtle loosening, low-grade infection or arthrofibrotic adhesions [1,16,22,35,50,52]. Sugimura et al. used metal-artifact-reduction MRI and power Doppler ultrasound to associate synovial and periarticular abnormalities with chronic post-TKA pain, supporting low-grade local tissue activity as a pain source even without gross implant failure [50]. Arthrofibrosis is itself a potent nociceptive generator, with dense capsular tissue and adhesions raising tension during motion and causing pain at extremes and sometimes at rest [10,29,52].

Peripheral nerve injury is common during TKA, particularly to the infrapatellar branch of the saphenous nerve, vulnerable during midline incision and medial parapatellar arthrotomy. Most patients experience only numbness, but a subset develop painful neuroma or neuropathic pain—burning, electric-shock pain, paresthesia, dysesthesia and mechanical allodynia [104,105]. Neuroma pain is focal, reproducible by Tinel testing and temporarily relieved by diagnostic block, and selected patients benefit from neuroma-directed surgery [105]. This matters because it can produce pseudo-stiffness: the patient avoids motion that stretches a painful or sensitized nerve even though passive joint motion is mechanically available. The common peroneal nerve should also be considered in marked valgus knees or after substantial lateral soft-tissue release, where correction may produce stretch, ischemic or direct injury with foot drop, neuropathic pain, weakness and delayed rehabilitation; in a systematic review of valgus TKAs, common peroneal nerve palsy occurred in 26 of 1397 knees (1.9%), although evidence that prophylactic peroneal nerve release prevents palsy remains limited [106].

Neuropathic-like pain is not limited to discrete neuroma. painDETECT and DN4 studies identify likely or possible neuropathic features in a meaningful subset of persistent post-TKA pain, associated with greater pain interference, worse function and poorer mental health [16,51,107,108]. CRPS is an uncommon but severe neuropathic–autonomic state—pain disproportionate to injury, allodynia, swelling, vasomotor or sudomotor change, temperature asymmetry, motor dysfunction and trophic change [23,109,110]—that can produce profound pain-limited stiffness despite minimal structural abnormality, demonstrating how severe pain and apparent stiffness arise from altered pain processing rather than implant failure.

### 5.4. Central and Nociplastic Pain Mechanisms

Central sensitization helps explain why some patients have high pain after TKA despite acceptable implant position, absence of infection and limited local pathology. It refers to enhanced responsiveness of central nociceptive pathways, reduced descending inhibition and pain hypersensitivity that outlasts tissue healing, so the knee stays painful not only from local input but because the nervous system is primed to amplify pain [16,23,40,42].

Quantitative sensory testing (QST) shows that many TKA candidates have preoperative widespread hyperalgesia, enhanced temporal summation and impaired conditioned pain modulation (CPM)—evidence of altered central processing that predicts less complete pain relief after arthroplasty [40,111,112,113,114,115,116]. A TKA-specific systematic review confirmed preoperative neuropathic-like pain and central sensitization as risk factors for chronic post-surgical pain [117], and nociplastic pain affects more than one third of hip and knee arthritis patients, predicting smaller functional gains and greater opioid use [118]. Such measures have been incorporated into prediction rules for chronic postsurgical pain [40]. The clinical correlate is the widespread or “poly-pain” phenotype: Wylde et al. found the number of other painful body sites strongly associated with persistent severe pain after TKA [16].

Neuroimaging supports the biological plausibility of central pain persistence: Barroso et al. identified structural and functional alterations in subcortical pain-processing regions in chronic post-TKA pain, consistent with broader evidence of subcortical and cortical changes in chronic pain [48]. Central mechanisms may pre-date surgery, be amplified by severe acute postoperative pain, or be maintained by ongoing nociceptive input, so that in a susceptible subset pain evolves from a knee-centric nociceptive problem into a widespread, centrally facilitated state in which revision directed at the knee alone is unlikely to help unless a clear mechanical or infective driver is also present [16,23,40,42,48].

### 5.5. Psychosocial and Behavioral Amplification

Psychosocial factors are not merely reactions to pain; they influence pain processing, rehabilitation behavior, sleep, activity and disability. Catastrophizing, anxiety, depression, fear of movement, poor sleep, low self-efficacy, unmet expectations, social stress and limited rehabilitation access can amplify nociceptive input and increase the risk that postoperative symptoms become chronic [2,4,5,6,7,14,16,41,42].

Pain catastrophizing is among the most consistent psychological correlates of persistent pain after TKA, and systematic reviews identify catastrophizing, high preoperative pain and pain in other body regions as important predictors of worse long-term pain [14,36]. Terradas-Monllor et al. linked early postoperative pain intensity, catastrophizing, fear of movement, anxiety, depression and maladaptive pain attitudes to chronic postsurgical pain [41], and Brander et al. similarly found preoperative pain and psychological distress predicted persistent pain and greater rehabilitation resource use [2,55].

Fear of movement provides a direct behavioral bridge between pain and stiffness: a patient who believes flexion will damage the implant may avoid end-range motion and guard during physiotherapy, initially producing pain-limited stiffness and, if persistent, capsular shortening, weakness and secondary contracture. Bhave et al. showed that some apparently stiff post-arthroplasty knees improve substantially with intensive rehabilitation, bracing and neuromuscular intervention rather than fixed arthrofibrosis [28], and cross-lagged analysis found reduced early knee movement predicted greater rest pain two weeks later, implicating acute-phase avoidance in prolonged pain [119].

Sleep is another amplifier: Owens et al. showed that maladaptive sleep–pain behaviors predicted insomnia and pain after TKA, with early insomnia mediating later pain [42]. Poor sleep increases pain sensitivity and reduces rehabilitation tolerance, while depression and anxiety further undermine recovery [4,5,16,55]. Expectations also matter—realistic expectations support engagement; whereas, unmet expectations and distrust are strongly associated with dissatisfaction and persistent pain [4,6,7,41].

Social and contextual factors shape whether these mechanisms resolve or persist: physically demanding work makes residual stiffness or anterior knee pain less tolerable, limited access to high-intensity rehabilitation or multidisciplinary pain care may let modifiable trajectories become chronic, and socioeconomic disadvantage compounds comorbidity, delayed care and poorer support [6,16,61,120]. Psychosocial factors are thus part of the causal and behavioral loop through which local tissue pathology becomes long-term disability.

### 5.6. Integrated Mechanistic Model

The mechanisms described above converge into a practical model: mechanical triggers such as malrotation, gap imbalance, patellofemoral overload, instability, impingement or overstuffing generate abnormal stresses, nociceptive pain and synovitis that, in susceptible knees (prior surgery, hemarthrosis, infection, severe preoperative stiffness or biological susceptibility), may evolve into arthrofibrosis with excessive scar formation, capsular contracture and loss of passive ROM [29,35,38,52]. Superimposed nerve injury, neuroma or CRPS introduces neuropathic pain, while pre-existing or postoperative central sensitization amplifies and generalizes it [16,23,40,48,51,104,105,107,108,109], and psychological distress, poor sleep, fear of movement and unmet expectations determine whether these processes are contained or spiral into chronic disability [2,4,5,6,7,14,41,42].

This integrated model has three major implications. First, no single mechanism explains all painful or stiff TKAs. Second, the dominant mechanism may change over time: early nociceptive pain may produce guarding; guarding may contribute to stiffness; stiffness may amplify nociceptive input; and persistent pain may become centrally sensitized. Third, effective treatment requires matching the intervention to the dominant modifiable driver. Mechanical malrotation requires mechanical correction; mature arthrofibrosis may require manipulation under anesthesia, arthrolysis or revision; neuroma requires nerve-targeted assessment and treatment; central sensitization requires multidisciplinary pain care; and pain-limited stiffness requires psychologically informed rehabilitation rather than reflex revision. This mechanistic framework provides the foundation for the risk-factor, phenotype, diagnostic and management sections that follow.

**Figure 2:**
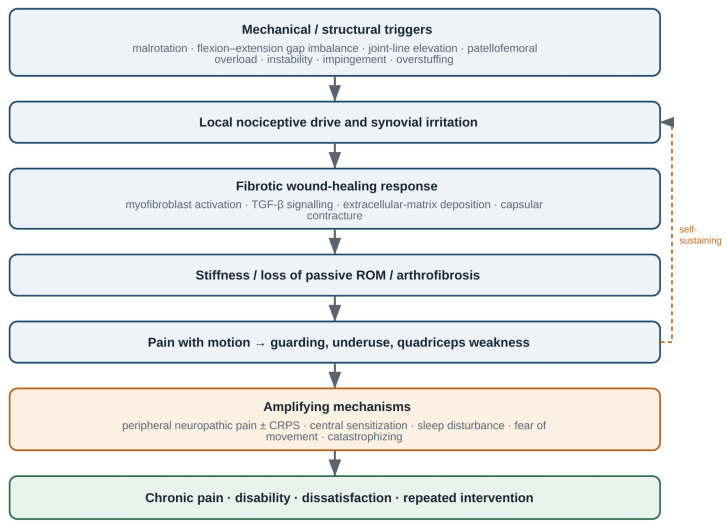
Integrated mechanism model of chronic pain and stiffness after total knee arthroplasty. The model emphasizes the self-sustaining feedback loop among nociceptive input, guarding, stiffness, inflammation and fibrosis, and central sensitization, in which each process can amplify the others.

This integrated mechanism model is summarized in Figure 2.

## 6. Risk Factors and Prediction

Chronic pain and stiffness after TKA arise from a layered accumulation of risk across the perioperative pathway: preoperative vulnerability, local joint disease, technical and anesthetic factors, and the early recovery trajectory. No single factor is determinative; mechanical, biological and psychological risks interact, and the same variable often influences both outcomes but with different weights [2,4,5,13,14,35,52,55]. Two broad tracks recur: a pain-dominant track, in which high preoperative pain, widespread pain and psychological distress predispose to CPSP and to pain-limited stiffness, and a stiffness-dominant track, in which severe preoperative ROM limitation, prior surgery, infection and deformity predispose to mechanical stiffness and arthrofibrosis. The most clinically actionable factors and early triggers are summarized in Table 3, grouped by clinical risk domain and perioperative phase, while the complete risk-factor matrix—reporting the direction and consistency of association for chronic postsurgical pain and for stiffness separately—is provided as Supplementary Table S1; the text highlights the strongest and most modifiable.

### 6.1. Preoperative Patient- and Joint-Related Factors

On the pain side, high preoperative pain intensity is among the most consistent predictors of worse 12-month pain [2,4,5,14,36,41], with rest or night pain particularly ominous [7], and widespread multi-site pain signaling central sensitization and severe persistent knee pain [14,16,36,40]. Catastrophizing is arguably the single most consistent psychological predictor of CPSP, clustering with depression, anxiety and kinesiophobia, while unmet expectations predict dissatisfaction even when objective scores are acceptable [4,6,14,41,55]. On the stiffness side, acquired idiopathic stiffness (AIS) is roughly three times commoner in women and doubles with BMI ≥ 30 kg/m^2^ [13,52], although modern optimization and rehabilitation may attenuate this historically reported risk [121]; severe preoperative ROM limitation (flexion < 50–60° or fixed flexion ≥ 20–30°) raises stiffness risk despite most knees still gaining motion [10,30,122,123,124], and prior surgery, post-traumatic or post-infectious knees, severe deformity and inflammatory arthropathy prime a profibrotic, scar-prone milieu [12,13,30,35,92,125,126,127,128,129]. Demographics cut across both tracks—younger age (<65 years) predicts dissatisfaction and MUA, and non-White race and lower income capture disparities in access and expectations [6,7,13,63]—while diabetes, higher ASA class and maladaptive sleep–pain behavior add further risk [4,13,27,42].

### 6.2. Perioperative and Surgical Factors

Surgical and anesthetic choices shape risk mainly through early pain control and joint mechanics. No single anesthetic technique prevents CPSP; because high-intensity acute pain is itself a key chronic-pain risk, multimodal regimens that deliver stable low early pain and enable rehabilitation are likely protective [14,23,40,41]. As in Section 5, the modifiable mechanical contributors are combined femoral–tibial internal rotation, joint-line elevation, abnormal tibial slope, flexion–extension gap mismatch, flexion-space overstuffing and unaddressed patellofemoral mechanics (rotation, tracking and, in selected designs, patellar resurfacing) [8,11,21,22,24,26,85]. Implant constraint, fixation mode and navigation or robotics show no consistent independent association once alignment is acceptable, although over-constraint with tight gaps increases stress and stiffness [1,8,24,35]. Soft-tissue handling matters: meticulous hemostasis, preservation of extensor-mechanism length and balanced releases reduce risk; whereas, difficult exposures and extensor-lengthening maneuvers (quadriceps snip, V–Y plasty, tibial tubercle osteotomy) increase stiffness, reoperation and residual pain [11,29,35,122,123,124].

### 6.3. Early Postoperative and Rehabilitation Factors

The early trajectory both predicts and shapes the chronic outcome. High pain at one week independently predicts CPSP at three and six months even after adjustment for catastrophizing and mood [40,41], and early ROM milestones are powerful: an arc < 50° at 2–4 weeks marks high risk for persistent stiffness and reintervention, and failure to reach ~90° flexion by 4–6 weeks usually triggers intensified therapy or MUA [10,12,37]. Early local complications—hemarthrosis, wound problems, low-grade PJI and venous thromboembolism—are potent triggers, with AIS commoner after early complications and readmissions [13,35,91]. Emerging insomnia and “yellow flags” (disproportionate distress, fear of end-range movement, low motivation, catastrophizing) mark the transition from acute nociception to a chronic trajectory and, if unrecognized, let pain-limited stiffness and CPSP consolidate [41,42]. Rehabilitation is the principal modifiable lever: confidence-building physiotherapy resolved more than 90% of complex motion problems without revision in one large series; whereas, poor access, low adherence and either under-use or premature over-use worsen outcomes [28,52,58,61,111,130].

### 6.4. Toward Risk Stratification and Prediction

Although many risk factors are well established, validated tools that combine them remain underdeveloped: most examine isolated predictors, use heterogeneous definitions, and assess pain or stiffness rather than both [13,14,36,52]. Useful prediction integrates three domains—baseline profiling (demographics, comorbidity, preoperative ROM, prior surgery, deformity and diagnosis with pain intensity and distribution, psychological distress, sleep and expectations) to flag patients needing counseling, prehabilitation, pain optimization or psychological support before surgery [4,5,13,14,36,40,41,42,55]; mechanistic profiling adding central-sensitization and fibrosis markers such as quantitative sensory testing, which improves CPSP prediction but belongs mainly to research and high-risk referral contexts [35,38,40,52,112,113,114,115,116]; and dynamic postoperative updating of pain, sleep, activity and ROM, reclassifying patients who develop severe pain, insomnia, flexion < 90° at six weeks or escalating fear of movement, whose interventions (intensified physiotherapy, bracing, early MUA, structured pain pathways) are time-sensitive [28,37,41,42,58,131,132]. Recent time-dependent modeling confirms that CPSP risk is dynamic rather than fixed, supporting repeated reassessment over a single preoperative label [133]. Crucially, stratification is valuable only insofar as it triggers action: structured pathways such as STAR improved HRQoL and reduced costs, so a usable model is judged by whether it improves patient-centered outcomes and prevents chronic disability, informing the phenotypes (Section 7), diagnosis (Section 8) and management (Section 9, Section 10 and Section 11) that follow.

## 7. Clinical Phenotypes of the Painful and/or Stiff TKA

A recurring message of this review is that the painful and/or stiff TKA is not a single entity but a spectrum of overlapping clinical phenotypes defined by the interplay of ROM profile, pain quality and distribution, mechanical status, and biopsychosocial context [2,10,18,35,52,55,67]. The six phenotypes below are heuristic groupings rather than rigid diagnostic boxes, and patients may migrate between them over time—an initially pain-dominant, guarded knee can evolve into a stiff, painful TKA with arthrofibrosis if early problems go unaddressed [10,41,52]. Their clinical value is not taxonomic precision but the discipline of identifying the dominant modifiable driver, so that treatment is matched to mechanism and both under-treatment of a correctable mechanical fault and over-treatment of a centrally driven or neuropathic problem are avoided. Their defining features, mechanisms and management directions are summarized in Table 4.

### 7.1. Pain-Dominant with Preserved ROM

These patients report troublesome pain despite a preserved arc (flexion ≥ 100–110°, contracture < 10–15°), no mechanical block, and often acceptable radiographs; the complaint is pain, not restriction [1,21,22]. Many harbor subtle mechanical sources that do not yet limit motion—patellofemoral overload or maltracking from an unresurfaced patella, malposition or internal rotation [21,22], low-grade synovitis or fat-pad/posterior-capsule impingement visible only on metal-artifact-reduction MRI [50], or mid-flexion micro-instability [11,24]—superimposed on a mixture of nociceptive, neuropathic and early central features [2,16,41,42,55]. The clinical lesson is twofold: a painful, well-moving knee should not be dismissed as “psychological”—subtle patellofemoral, rotational, synovial or neuroma sources must be actively sought [21,22,104,105]—yet central sensitization and distress are common and should be assessed and managed, and revision for pain without a clear mechanical target carries a high risk of persistent pain [1,23,35,58].

### 7.2. Stiffness-Dominant with Modest Pain

Here the complaint is restricted motion and functional loss, with pain absent or modest except at extremes; flexion is ~75–100° and/or contracture 10–20° with a firm end-feel [9,10,14]. Contributors include residual or recurrent capsular tightness after an initially stiff knee, mild-to-moderate arthrofibrosis with low resting nociceptive drive, extra-articular contracture (extensor shortening, hamstring or hip flexion contracture), and heterotopic ossification [30,52,89,90,91]. This phenotype illustrates that ROM can be function-limiting even when pain is modest; management emphasizes mechanical and rehabilitative strategies—high-intensity physiotherapy, static-progressive bracing and, where indicated, MUA or arthrolysis before revision [28,32,33,37]. Because pain is unobtrusive these patients may never reach a pain service, yet their participation, especially when young or working, is often profoundly affected [13,14].

### 7.3. Combined Severe Pain and Stiffness (“Stiff, Painful TKA”)

The archetypal failure pattern combines flexion often <75–80° (± contracture) with severe pain at extremes and often at rest or night, marked disability and near-universal dissatisfaction [3,10,18]. It usually reflects convergence of mechanical error (malposition, gap imbalance) and arthrofibrosis, sometimes compounded by low-grade infection and central sensitization [11,22,24,29,52,91,101]. This group is heavily overrepresented among reoperations and high-cost patients (Section 4) and almost always requires combined mechanical and pain-focused treatment—correction of malposition or imbalance and arthrolysis of fibrotic tissue, plus optimization of analgesia, sleep and psychological support [18,19,29,35,52]. Even after technically successful revision, ROM often remains 80–100°, but patients may still report satisfaction if pain is adequately reduced; conversely, neglecting psychosocial and sleep factors can leave persistent CPSP despite improved mechanics [3,19,23,42].

### 7.4. Neuropathic-Dominant (Nerve Injury, Neuroma, CRPS)

This phenotype is marked by burning, electric-shock, pins-and-needles or numb sensations with mechanical or thermal allodynia, often with focal tenderness over a nerve course, and ROM preserved or limited mainly by guarding [16,104,105,107,108,109]. The infrapatellar branch of the saphenous nerve is especially vulnerable: symptomatic neuromas are reported in selected series, more frequently after revision, with Tinel-positive shock-like pain relieved by diagnostic block and improved by resection or relocation [105]. Screening tools identify likely neuropathic pain in ~6% of the whole TKA population and many more with “possible” neuropathic features, correlating with worse pain and Oxford scores [16,51,107]. CRPS is the extreme, with disproportionate pain, allodynia, edema, vasomotor/sudomotor and trophic change, and profound guarding-related stiffness despite minimal structural pathology [23,109]. Recognition is decisive: these knees respond poorly to revision but may improve with neuropathic pharmacotherapy, carefully dosed physiotherapy, psychological therapy and, where appropriate, neuroma surgery or regional intervention; stiffness here is pain-limited and should not be met with aggressive arthrolysis absent demonstrated structural pathology [23,28,104,105,109].

### 7.5. Centrally Sensitized/Widespread Pain

Dominated by widespread pain and central sensitization, the replaced knee is one of several painful sites, accompanied by fatigue, poor sleep and low mood, with pain poorly correlated to local findings and abnormal quantitative sensory testing (QST) [16,23,40,42,48,113,114,115,116]. The number of other painful sites is among the strongest predictors of severe persistent knee pain (a significant, independent association), preoperative widespread hyperalgesia and impaired conditioned pain modulation predict poorer relief, and MRI shows thalamic, basal-ganglia and limbic alterations—together describing a nociplastic pattern that joint replacement alone cannot cure [16,40,48,113,114,115,116]. ROM is variable: some maintain near-normal motion with high pain, others develop pain-limited stiffness with a soft end-feel and greater motion under anesthesia, true arthrofibrosis being uncommon unless other risks coexist [28,35,52]. Management is system-level—multidisciplinary pain care, CBT or acceptance-based therapy, psychologically informed physiotherapy and graded activity—and revision without a clear mechanical target should be avoided as it risks worsening pain [23,35,58,111,130].

### 7.6. Arthrofibrosis with Mechanical Block but Mild Pain

A minority of arthrofibrotic knees present with marked restriction (flexion < 75° and/or contracture ≥ 20°), a firm abrupt end-feel and only modest resting pain, the patient reporting that “the knee will not bend or straighten” [10,29,52]. The substrate is dense capsular fibrosis and adhesions—driven by persistent myofibroblast activity, aberrant TGF-β signaling and immune infiltration—with relatively muted nociception once synovitis settles, sometimes with heterotopic bone bridging [38,52,89,93,94,97,101]. The key discriminator from pain-limited stiffness is that passive ROM remains restricted even under anesthesia with an abrupt end-feel; in such mature, established mechanical block isolated forceful manipulation yields little and carries fracture risk, so arthrolysis or revision is usually preferred [10,28,29]. These patients benefit most from mechanically focused treatment—early MUA, arthroscopic or open arthrolysis, or revision with extensive release ± greater constraint—and, because baseline pain is modest, satisfaction depends heavily on achieving ROM gains that cross functional thresholds (a rise from 60° to 80° may still leave real disability) [9,10,29,32,33,52].

### 7.7. Special Populations

Certain groups cluster within particular phenotypes. Preoperatively stiff or ankylosed knees (flexion < 50–60°, contracture ≥ 20–30°, often after infection, trauma, inflammatory disease or multiple surgeries) achieve substantial gains but end with lower final ROM and higher complication rates, frequently transitioning to stiffness-dominant or stiff-painful patterns unless meticulous balancing, joint-line restoration, early intensive rehabilitation and recognition of profibrotic risk are in place [13,29,30,35,122,123,124,134,135,136]. Revision TKA patients sit at the intersection of the stiff-painful and centrally sensitized phenotypes: combined malposition, arthrofibrosis, bone loss and extensor compromise—sometimes with low-grade infection and frequent neuropathic or central features—mean pain often improves after correction while ROM gains are modest, and repeated revision without a mechanical target yields diminishing returns and escalating cost [3,19,23,35,59,137,138]. Cultural and health-system context shapes how phenotypes present and are managed: Western registries report ~10–20% dissatisfaction (contemporary estimates 7–14%) driven by pain, stiffness and unmet expectations rather than radiographic failure [6,7,64], fragmented services can entrench central and neuropathic phenotypes [58,61], and differences in rehabilitation access, work demands and social support determine how heavily a given phenotype affects a patient’s life [83,84,120]. Recognizing a patient’s phenotype—and the contexts that shape it—is the basis for the phenotype-informed diagnostic algorithm (Section 8) and the prevention and treatment strategies (Section 9, Section 10 and Section 11) that follow.

## 8. Diagnostic Work-Up and Phenotype Assignment

**Figure 3:**
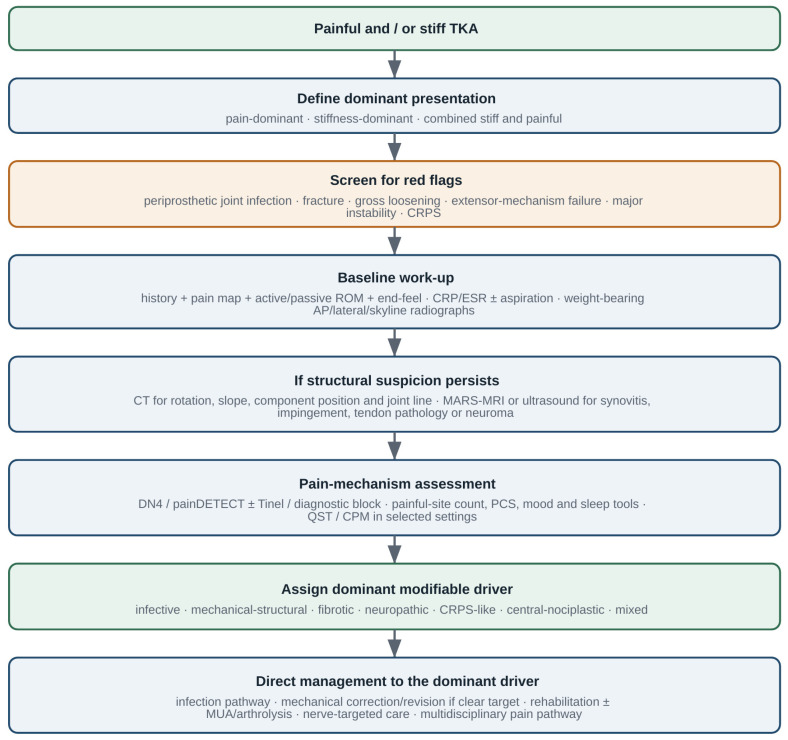
Phenotype-based diagnostic algorithm for the painful and/or stiff TKA.

The aim of diagnosis is not merely to “find the lesion” but to map the patient onto one or more of the phenotypes of Section 7 and identify which drivers are realistically modifiable, since mechanical, biological and psychosocial mechanisms almost always coexist [2,10,18,35,52]. A pragmatic work-up proceeds in layers—establishing the nature and time course of symptoms, excluding urgent problems, evaluating mechanics and fibrosis, and phenotyping pain—and is summarized as an integrated algorithm in Figure 3.

### 8.1. Practical Phenotype Assignment in Routine Practice

Phenotype allocation (Table 4) is intended as a bedside exercise built from a defined minimum dataset rather than a research protocol. At outpatient review this comprises goniometric active and passive range of motion (ROM) with end-feel; weight-bearing anteroposterior, lateral and skyline radiographs; assessment of patellar tracking and stability; inflammatory markers, with aspiration where infection is plausible; a pain map and painful-site count; neuropathic screening (DN4 or painDETECT); and brief psychological and sleep screening (Pain Catastrophizing Scale, a kinesiophobia item, and a mood and insomnia check). The individual tools are described in Section 8.2, Section 8.3, Section 8.4, Section 8.5 and Section 8.6; the purpose here is to combine them into a working phenotype [2,16,22,35,40,41,42].

Assignment is best made serially rather than at a single time point: early review separates pain-limited from mechanical restriction and flags high-risk trajectories, the three-month mark provides the conventional CPSP threshold for formal phenotyping, and re-assessment is warranted before any escalation to MUA, arthrolysis or revision [10,13,41,42]. Because most patients occupy more than one domain, a simple priority rule applies. Infection, fracture and gross mechanical failure are excluded first; a clear, correctable mechanical target—for example CT-confirmed component malrotation—is then treated as the dominant modifiable driver, but only after pain and psychosocial phenotyping, since a high neuropathic or central burden mandates simultaneous pain-pathway management and tempers the expected benefit of mechanical correction. Where no correctable mechanical target exists and the dominant phenotype is neuropathic, central or psychosocial, revision is avoided [16,23,35,40,109].

For example, a patient seen four months after primary TKA with flexion of 85°, a painDETECT score consistent with possible neuropathic pain, high pain catastrophizing and CT-confirmed combined internal rotation of the femoral and tibial components occupies the combined stiff-and-painful and neuropathic domains simultaneously. The CT abnormality represents a potentially correctable mechanical target only if it is clinically concordant with the pain pattern, patellofemoral findings and stiffness profile, and after infection has been excluded. Management should therefore proceed in parallel: confirm mechanical concordance and revision suitability while initiating neuropathic-pain treatment, psychologically informed rehabilitation and expectation-setting. If revision is ultimately undertaken, it should be framed as correction of the mechanical driver, not as a stand-alone cure for neuropathic or centrally amplified pain [22,23,40,109].

### 8.2. History and Physical Examination

History and examination remain the most informative part of the work-up and frequently distinguish mechanical problems from neuropathic and centrally mediated pain [1,16,22,23,35,52]. Pain location orients the differential—anterior or peripatellar pain worse on stairs and chair rise suggests patellofemoral overload, maltracking or rotational malalignment; joint-line pain suggests asymmetric loading or focal loosening; posterior pain suggests capsular contracture or arthrofibrosis; focal incisional tenderness suggests cutaneous nerve injury or neuroma; and pain beyond the knee signals widespread, centrally sensitized pain [10,11,16,22,23,105]. Quality adds mechanism: aching activity-related pain reflects nociception, sharp catching pain reflects impingement or maltracking, and burning, electric or allodynic pain reflects neuropathic generators, with a swollen “on-fire” limb raising CRPS [1,23,51,105,107,108,109]. Temporal pattern matters—pain that never improves suggests unresolved mechanical fault or early central sensitization, pain after a pain-free interval suggests late infection, wear or instability, and night/rest pain points to high nociceptive drive or sensitization [7,10,14,35,42]. Inquiry into other painful sites, sleep and mood is essential, since early insomnia and catastrophizing predict and are modifiable contributors to CPSP [2,41,42].

Examination begins with active and passive ROM (goniometer, compared with the contralateral knee), documenting flexion, contracture and—critically—end-feel: a soft, pain-limited end-feel with a large active–passive discrepancy indicates guarding amenable to analgesia and psychologically informed physiotherapy; whereas, a firm, abrupt end-feel restricted equally awake and under anesthesia indicates true mechanical contracture from arthrofibrosis, heterotopic ossification or extra-articular tethering [10,28,29,38,52]. Arthrofibrosis is a diagnosis of exclusion—infection, gross malposition, instability and extra-articular deformity must be ruled out first—and intra-articular fibrosis (global, symmetric, hard stop) must be distinguished from extra-articular causes such as malunion, extensor-mechanism shortening or hip flexion contracture (directional or posture-dependent) [29,35,38,52,91]. Gait and task-specific testing (sit-to-stand, stairs, attempted kneeling), timed tests and PROMs place findings in functional context and quantify impact over time [4,5,10,52]; serial measurement against a knee’s own early trajectory or the contralateral side often identifies an evolving problem before it becomes fixed. Throughout, red flags demand expedited action: new or rest/night pain with effusion, warmth or wound problems (PJI, which may present mainly as stiffness with near-normal markers); recurrent giving-way with examinable laxity (instability); disproportionate pain with vasomotor, sudomotor and trophic change (CRPS); and burning, allodynia and a Tinel-positive focus along the IBSN (neuroma)—the last two responding poorly to prosthesis-directed surgery [11,23,24,35,91,102,105,109].

### 8.3. Examination Under Anesthesia

Examination under anesthesia (EUA) is the most direct way to separate pain-limited from true mechanical stiffness and should be considered when clinic findings are equivocal: a persistent active–passive ROM discrepancy, an unclear or inconsistent end-feel, suspected guarding-dominant restriction, and as a deliberate pre-MUA or pre-revision assessment [27,28,33,52]. With the patient fully relaxed, flexion, extension, end-feel and patellar mobility are documented and interpreted against the awake examination. A substantial gain in passive ROM under anesthesia indicates a predominantly pain-limited or guarding-driven pattern likely to respond to analgesic optimization, psychologically informed physiotherapy and graded exposure rather than to forceful intervention. Passive ROM that remains restricted with a firm or abrupt end-feel indicates true mechanical restriction from arthrofibrosis, heterotopic ossification, component-related block or extra-articular tethering. In early or immature stiffness this may support carefully selected MUA once infection, fracture, instability and malposition are excluded; in mature contracture, heterotopic ossification, extra-articular tethering or component malposition, isolated forceful manipulation is less likely to succeed and arthrolysis or revision may be more appropriate. Intermediate gains suggest a mixed phenotype requiring combined mechanical and pain-focused management. The passive arc achieved under anesthesia should be recorded as a baseline that informs subsequent MUA, arthrolysis or revision decisions [10,28,29,32].

### 8.4. Laboratory Tests and Joint Aspiration

Laboratory testing primarily excludes infection. Serum C-reactive protein and erythrocyte sedimentation rate are first-line, but normal values do not exclude low-grade PJI, so most authors advocate a low threshold for aspiration in any painful or stiff TKA with concerning features [35,91,102]. Synovial fluid cell count and differential, with prolonged aerobic and anaerobic culture, can reveal indolent organisms (Cutibacterium acnes, coagulase-negative staphylococci) that present with stiffness rather than sepsis, and adjunct biomarkers such as α-defensin or leukocyte esterase aid PJI diagnosis; results are best interpreted within validated frameworks such as the Musculoskeletal Infection Society and International Consensus Meeting criteria rather than from any single test [35,91,101,102]. Importantly, arthrofibrotic synovial fluid typically lacks the leukocytosis and inflammatory markers of infection, so a “quiet” aspirate in a stiff, painful knee should not be assumed benign but may reflect a fibrotic, immune-mediated process; at revision, multiple intra-operative tissue cultures remain the reference standard whenever stiffness or unexplained pain is the indication [91,101,102].

### 8.5. Imaging

Imaging should be driven by specific clinical questions rather than used indiscriminately (Table 5). Weight-bearing radiographs are first-line and exclude gross malposition, loosening, fracture and severe HO; CT is the modality of choice for component rotation—measured against the surgical or clinical transepicondylar axis and the tibial tubercle, with combined femoral and tibial internal rotation the finding most associated with anterior knee pain—and frequently supplies the mechanical target for revision; metal-artifact-reduction MRI and ultrasound are used selectively for soft-tissue pathology, synovitis and neuroma; and nuclear or hybrid imaging is reserved for diagnostic dilemmas after other tests are inconclusive [11,22,24,35,39,50,89,139]. Dynamic fluoroscopy and instrumented motion analysis are not routine but can unmask patellar maltracking, mid-flexion instability or posterior impingement and quantify compensatory strategies in selected complex or pre-revision cases [10,35,52].

### 8.6. Pain Phenotyping and Psychological Assessment

Beyond structural evaluation, systematic assessment of pain mechanism and psychosocial context is essential to avoid futile surgery in neuropathic or centrally driven cases [2,16,23,40,41,42]. Neuropathic screening tools (painDETECT, DN4, LANSS) identify likely neuropathic pain in roughly 6% of TKA patients and many more with possible features, flagging candidates for neuropathic pharmacotherapy or nerve-targeted intervention and those at higher risk of poor response to mechanical surgery [16,51,107,108]. Psychological scales should be used proactively—the Pain Catastrophizing Scale, a kinesiophobia measure and brief mood scales (HADS, PHQ-9, GAD-7) capture the pain-related psychological distress that strongly predicts CPSP and creates an early window for education and psychological intervention [4,14,16,41]. In specialist settings, quantitative sensory testing and conditioned pain modulation provide objective indices of central sensitization and deficient descending inhibition that independently predict poorer pain relief, complemented by sleep and activity monitoring [40,42,48,113,114]. These tools rarely change the diagnosis alone but are decisive when a decision about further surgery hinges on disentangling mechanical from central drivers: with only equivocal mechanical findings, marked central sensitization, widespread pain, high catastrophizing or unaddressed neuropathic features weigh strongly against further prosthesis-directed surgery and toward a multidisciplinary pathway; whereas, their relative absence alongside a clear mechanical target supports targeted correction [16,23,35,40,109].

### 8.7. Integrated Diagnostic Algorithm

Bringing these elements together yields a phenotype-guided, “mechanics-first then mechanisms-of-pain” algorithm (Figure 3) [10,11,22,23,35,39,52]. After confirming the timeline and screening for red flags, baseline radiographs and inflammatory markers (with aspiration if PJI is suspected) are obtained; anterior knee pain with normal radiographs prompts CT for rotation, and otherwise unexplained pain prompts selective MARS-MRI or ultrasound, before pain and psychosocial phenotyping refine the picture. For stiffness, the additional pivot is separating pain-limited from true mechanical restriction (active versus passive ROM, end-feel, EUA if needed), since the former responds to intensified analgesia, psychological support and high-intensity physiotherapy while the latter requires mechanical solutions—and severe early stiffness (ROM < 90°, especially <50°) carries a high risk of fixed contracture and may warrant early MUA once infection and malposition are excluded [10,13,28,32,37,52]. The synthesis assigns one or more dominant etiologic domains, each with a primary treatment direction: mechanical–structural → targeted correction or revision; fibrotic → high-intensity rehabilitation, MUA, arthrolysis; infective/inflammatory → organism-directed therapy and appropriate surgery; neuropathic → nerve-targeted and multidisciplinary pain care; and central/nociplastic → structured pain pathways, psychologically informed physiotherapy and graded exercise with at most a supportive surgical role [23,35,38,52,105,109]. Because most patients occupy more than one domain, the purpose of classification is not a single label but to clarify which domain is most modifiable and should be targeted first, and where further surgery risks more harm than benefit—principles that carry into the prevention (Section 9) and management (Section 10 and Section 11) strategies that follow.

## 9. Prevention and Early Intervention Across the Perioperative Pathway

Prevention of chronic pain and stiffness after total knee arthroplasty (TKA) is best understood as a perioperative pathway rather than a single intervention. Because persistent symptoms arise from interacting mechanical, fibrotic, neuropathic, central and psychosocial mechanisms, preventive care must begin before surgery, continue through technical execution and acute pain control, and extend into early rehabilitation and structured trajectory monitoring [23,35,47,52]. Two principles apply. Prevention should be risk-matched—a patient with widespread pain, catastrophizing and insomnia needs a different pathway from one with severe contracture and multiple prior operations—and dynamic, because some apparently low-risk patients declare risk through severe acute pain, early insomnia, hemarthrosis, wound problems, delayed range-of-motion (ROM) recovery or fear-avoidant behavior [10,12,13,32,33,41,42,59]. Baseline risk profiling must therefore be coupled with early postoperative trajectory monitoring.

### 9.1. Preoperative Risk Stratification and Optimization

Preoperative assessment should identify patients at increased risk of chronic postsurgical pain (CPSP), stiffness, arthrofibrosis or dissatisfaction—not to exclude them, but to inform consent, align expectations and plan support. The relevant risk factors are detailed in Section 6; preventively, the priority is to translate them into action. Patients with widespread or disproportionate pain, central-sensitization features, catastrophizing, depression, anxiety or maladaptive sleep–pain behavior should be counseled that TKA may reduce joint nociception without normalizing the broader pain system, and considered for psychologically informed rehabilitation, sleep intervention or pain-specialist input, with brief tools (Pain Catastrophizing Scale, PHQ-9, GAD-7, insomnia and kinesiophobia measures) flagging them efficiently [2,14,16,23,36,40,41,42,55,140,141,142]. Those with severe stiffness, ankylosis, multiple prior operations or post-infectious or post-traumatic pathology should be told that pain relief may be substantial but final ROM is likely lower and complication risk higher [9,13,30,35,122,123,124,125,134,135,136,143,144].

Prehabilitation should be selective rather than generic: extension, flexion, quadriceps activation, gait and ROM-milestone education in stiffness-prone patients; pacing, graded activity, sleep optimization and pain education in pain-prone or centrally sensitized patients [41,42,111,145,146,147,148,149]. In high-catastrophizing candidates, home-based multimodal physiotherapy combining pain neuroscience education, coping-skills training and exercise is a risk-matched option [150]. Chronic opioid use should be identified early and, where feasible, rationalized with primary care or pain medicine to reduce opioid-induced hyperalgesia and difficult postoperative pain control [151,152,153,154,155,156,157]. Expectation management is itself preventive: patients should understand that TKA usually improves pain and function but does not guarantee a normal-feeling knee, deep flexion or painless kneeling, with goals individualized to baseline ROM, phenotype and activity demands [6,7,64,65,78].

### 9.2. Intraoperative Surgical Strategies

Intraoperative prevention reduces the mechanical and biological triggers of pain, synovitis and fibrosis. The mechanisms by which malrotation, gap imbalance, joint-line elevation, patellofemoral overstuffing and instability generate symptoms are detailed in Section 5; the preventive corollaries are accurate component positioning, balanced flexion and extension gaps, restoration of joint line and tibial slope where possible, stable but not over-constrained soft tissues, optimized patellofemoral mechanics and meticulous hemostasis [8,11,22,24,35,52,158]. In particular, femoral and tibial internal rotation and patellofemoral overstuffing should be avoided, appropriate patellar thickness and tracking maintained, and lateral release used selectively [21,22,159,160,161,162,163,164,165,166,167,168,169,170,171,172]. Component oversizing, excess polyethylene and flexion-space overstuffing should be avoided, especially in smaller or preoperatively stiff knees [8,26]; joint-line elevation can disturb collateral isometry, reduce quadriceps efficiency and cause mid-flexion instability and anterior knee symptoms [11,24,85].

Complex stiff knees require planned exposure and release rather than forceful manipulation. Ankylosis, severe contracture, prior infection or multiple previous operations may require posterior capsular release, osteophyte removal, extensile exposure, tibial tubercle osteotomy or increased constraint to achieve a stable functional arc; because aggressive exposure also increases tissue trauma and may promote fibrosis, it should be used deliberately, with meticulous hemostasis and a clear rehabilitation plan [29,30,31,35,52,122,123,124,134,135,136,143,144].

### 9.3. Anesthesia, Analgesia and Early Mobilization

Acute pain control is preventive because severe early pain promotes guarding, sleep disruption, reduced rehabilitation and central sensitization [14,23,41,42]. The aim is balanced analgesia that reduces pain and opioid exposure while preserving quadriceps function and enabling safe early mobilization [173,174,175,176,177,178]. Multimodal systemic analgesia should prioritize non-opioid agents when safe—acetaminophen and NSAIDs or COX-2 inhibitors—with opioids reserved for short-term rescue, and gabapentinoids individualized against sedation, dizziness, falls and uncertain CPSP prevention [173,179,180]. With preoperative widespread pain, opioid tolerance or neuropathic features, early pain-medicine input is more useful than indiscriminate opioid escalation. Regional techniques should support function: adductor canal blocks and local infiltration analgesia preserve ambulation; whereas, femoral nerve blocks give strong analgesia but may impair quadriceps activation [173,181,182]. The optimal strategy allows repeated active ROM, full-extension work and early walking without excessive motor block or sedation, with cryotherapy, compression and reassurance as supportive adjuncts [183,184,185].

### 9.4. Early Postoperative Rehabilitation and Trajectory Monitoring

Early rehabilitation should integrate extension, flexion, quadriceps activation, gait, swelling control and functional tasks rather than treating flexion alone as the endpoint. Full extension should be pursued early, because flexion contracture increases quadriceps demand, alters gait and becomes hard to reverse, while flexion progresses toward individualized milestones [9,10,52,53,146]. Monitoring should track ROM, active–passive discrepancy, end-feel, pain, swelling, sleep, gait and adherence; failure to reach approximately 90° flexion by 4–6 weeks, or plateauing below functional ROM despite adequate therapy, should prompt reassessment and escalation rather than passive reassurance, and a total arc < 50° at 2–4 weeks marks a rare but very high-risk group requiring urgent review [10,12,32,33,37,131,132].

Behavioral yellow flags should be weighed alongside ROM: persistent high pain, escalating analgesic use, insomnia, catastrophizing, fear of movement, avoidance of end-range work and beliefs such as “movement will damage the implant” signal risk for CPSP and pain-limited stiffness [2,16,41,42,55]. Such patients need analgesic optimization, reassurance, graded exposure and psychologically informed physiotherapy rather than being told to “push harder”. Continuous passive motion should not replace active rehabilitation—trials suggest at most small short-term flexion gains—though it may have a selective role after MUA or arthrolysis [146,186,187,188]. Home-based, supervised and telerehabilitation can all work when structured and monitored, with intensity matched to risk [189,190,191]. Digital tools and wearables may flag declining activity, plateaued pain or a poor ROM trajectory but should trigger clinical action rather than passive data collection [192,193,194].

### 9.5. Early Management of Emerging Stiffness or Troublesome Pain

When early stiffness or persistent pain emerges, the first response is structured reassessment: infection, wound problems, hemarthrosis, fracture, gross instability, extensor dysfunction, complex regional pain syndrome (CRPS) and major malposition must be excluded when suspected, and the clinician should determine whether the problem is predominantly pain-limited, fibrotic, mechanical or mixed [35,52,91,101,102,103,109]. Pain-limited stiffness—large active–passive discrepancy, soft end-feel, high fear, improvement with analgesia—is treated with analgesic optimization, swelling control, graded exposure, reassurance and psychologically informed rehabilitation, since forceful escalation may worsen symptoms if the pain mechanism is uncontrolled [23,28,41,42,52,109]. Emerging fibrotic or mechanical stiffness should trigger time-bounded intensified non-operative care—more frequent supervised physiotherapy, prolonged low-load stretching, capsular and patellar mobilization, static-progressive or dynamic bracing and selective anti-inflammatory strategies—escalating if ROM plateaus below functional thresholds despite adherence [28,146,149,185,195,196,197,198]. Observational data link perioperative agents such as celecoxib and dexamethasone to lower subsequent MUA or lysis rates, but a randomized trial of adjuvant anti-inflammatory medication after MUA did not improve ROM [138], so they remain investigational [199].

Early MUA occupies the boundary between prevention and treatment, most appropriate for function-limiting stiffness—commonly flexion < 90–95° at 6–12 weeks despite adequate analgesia and intensive therapy—after exclusion of infection, fracture, gross malposition, instability and CRPS; the central message is timing, since flexion gains are generally greater when MUA is performed within approximately three months, though selected later cases may still benefit [32,33,37,131,132,200]. Technique and outcomes are detailed in Section 10. Troublesome pain at around three months should likewise trigger structured, phenotype-based assessment rather than reassurance alone: the STAR pathway shows that proactive identification and multidisciplinary management of problematic post-TKA pain is feasible and economically promising compared with fragmented usual care [58,61,82].

**Figure 4:**
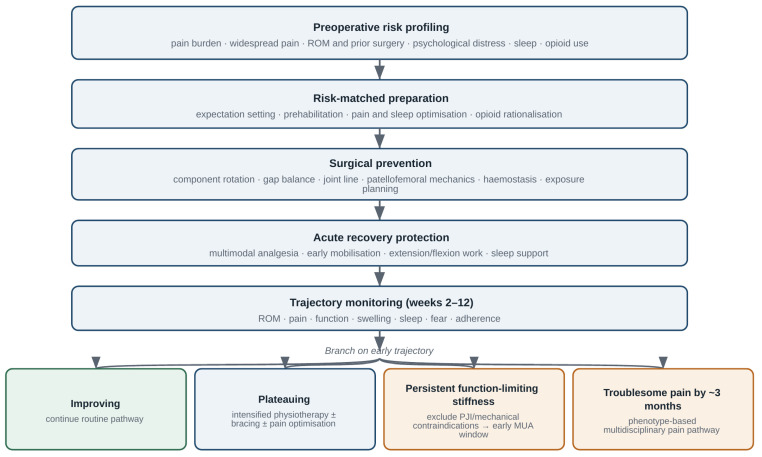
Phenotype-based prevention and early-intervention pathway after total knee arthroplasty.

The resulting phenotype-based prevention and early-intervention pathway is summarized in Figure 4.

## 10. Management of Established Stiffness and Arthrofibrosis

Management of established stiffness after TKA should be phenotype-guided, stepwise and multidisciplinary, matched to the dominant modifiable driver. The key question is not whether the knee is stiff but why, since pain-limited guarding, immature fibrosis, mature arthrofibrosis, malposition, gap imbalance, patellofemoral overstuffing, instability, heterotopic ossification, infection or extra-articular tethering can produce similar range-of-motion deficits yet require different strategies [13,28,32,33,34,35,38,52].

Once stiffness is established—typically a restricted arc impairing daily activities, often flexion < 90° and/or a flexion contracture > 10°, with variable pain—management shifts from prevention to salvage, most effective when matched to the dominant phenotype and substrate identified during diagnosis (Section 7 and Section 8): pain-limited and centrally driven restriction calls for analgesic, rehabilitative and psychological strategies; whereas, true mechanical contracture from mature fibrosis, malposition or extra-articular tethering calls for procedural escalation [32,33]. A stepwise pathway is generally advocated—from optimized non-operative care through early MUA, arthroscopic and open arthrolysis with or without limited component exchange, to full revision when a mechanical error is present—each step matched to substrate and summarized with typical ROM gains in Table 6.

### 10.1. Non-Operative Management

Non-operative care remains the foundation even in established stiffness, maximizing ROM and strength and reducing pain so that invasive procedures can be avoided or undertaken from a better baseline [28,146]. The cornerstone is structured, high-intensity rehabilitation—frequent supervised end-range stretching with therapist overpressure, joint and patellar mobilization, progressive (often eccentric, closed-chain) strengthening and functional retraining under good analgesia. In Bhave’s series of 118 problematic hip and knee arthroplasties, an individualized program of intensive physiotherapy, neuromuscular techniques and selective procedures such as peroneal nerve release avoided major reconstruction in 92% [28,201,202]. Static-progressive and dynamic stretch orthoses, which hold the joint at end range and exploit stress relaxation, give clinically meaningful gains—typically 10–25°—with high satisfaction, very low complications and applicability even after failed MUA or surgery [196,201,202,203,204,205]. Instrument-assisted soft-tissue mobilization and neuromuscular electrical stimulation are reasonable low-risk adjuncts; pharmacological agents (NSAIDs or COX-2 inhibitors, short corticosteroid courses for flares) are supportive, and antifibrotic agents targeting TGF-β or collagen cross-linking remain experimental [146,195,198,206,207].

### 10.2. Manipulation Under Anesthesia

MUA is pivotal—minimally invasive, inexpensive and capable of substantial ROM gains in selected patients, particularly before dense adhesions mature [32,33,132,208]. The usual indication is failure to achieve >90° flexion within 6–12 weeks despite compliant rehabilitation, with a plateau and a firm (rather than pain-limited) end-feel; contraindications include infection, gross malalignment or instability, component malposition, high fracture risk and severe CRPS [33,63]. Gradual controlled flexion force ruptures suprapatellar, notch and posterior-capsule adhesions, followed by patellar mobilization in extension, with immediate rehabilitation and analgesia (often epidural or peripheral catheters) to prevent re-adhesion [63]. Timing is the most critical variable: final flexion is significantly higher when MUA is performed within approximately three months, and most reviews support an early 6–12-week window [33,208]. Across the largest syntheses (e.g., Gu: 22 studies, 1488 patients), MUA increases the total arc by roughly 30–47°, with most patients reaching functional arcs > 90° and a failure rate around 6% [32,63,132]. Predictors of needing MUA include limited preoperative ROM, younger age and prior surgery, while very limited arcs (<60°), late presentation, malposition or pain disproportionate to mechanics respond poorly and tend to progress to arthrolysis or revision, about 17% needing further intervention in a recent 227-knee series [12,32,33,209,210,211,212,213]. Reported MUA outcomes should also be interpreted in light of technical heterogeneity: anesthetic technique, depth of muscle relaxation, use of epidural or peripheral catheter analgesia to facilitate post-MUA rehabilitation, speed and magnitude of applied force, patellar mobilization strategy and concurrent corticosteroid injection vary across centers and may contribute to the wide range of reported ROM gains and complications. Complications—supracondylar fracture, tibial tubercle avulsion, patellar tendon rupture and hemarthrosis—are rare with controlled, gradual force, and post-MUA radiographs are advisable to exclude occult fracture [33,63].

### 10.3. Arthroscopic Arthrolysis

Arthroscopic lysis suits patients in whom MUA has failed or the contracture is more mature, especially an isolated flexion deficit with preserved extension and well-positioned components [33]. It targets fibrous tissue in the suprapatellar pouch, gutters and notch, with posterior cruciate release in cruciate-retaining designs and notchplasty for impinging osteophytes, while posterior capsular release through posteromedial and posterolateral portals is more demanding [214,215]. Series report ROM gains of roughly 25–42° with 73–88% success, best with focal adhesions and least effective for global or extension-dominant stiffness [216,217,218,219]. Outcomes broadly match MUA in suitable patients, trading greater invasiveness for direct visualization and focal treatment, and—as with MUA—success depends on rigorous early rehabilitation; recent data support its use in selected post-TKA arthrofibrosis, patellofemoral impingement and mixed-contracture phenotypes with careful selection [33,216,220]. Individual manipulation, arthrolysis and revision series are summarized in Supplementary Table S2.

### 10.4. Open Arthrolysis and Limited Revision

When stiffness is severe, longstanding, or associated with extra-articular contracture or component issues that cannot be addressed arthroscopically, open arthrolysis—sometimes with polyethylene exchange—permits circumferential capsular and extra-articular release [215,221]. Reported gains span roughly 18–38° with 41–83% success but are heterogeneous, and isolated insert exchange performs poorly, so the procedure must be tailored to the pathology rather than applied indiscriminately [216,222]. Severely stiff knees often require extensile exposure: the quadriceps snip is quick and avoids extensor lengthening, V–Y turndown gives more lengthening at the cost of extensor weakness, and tibial tubercle osteotomy offers excellent exposure but risks non-union, migration and extensor lag [31]. Open arthrolysis suits well-fixed, well-aligned, soft-tissue-dominant stiffness, whereas clear malposition, wear, instability or maltracking favors revision; patients should be counseled that the goal is a 90–110° arc rather than normal motion, that pain may persist and rehabilitation is intensive, and that arthrodesis is rarely required [3,9,19,29,216].

### 10.5. Revision Arthroplasty

Revision is the final step, reserved for stiffness driven by correctable mechanical error—component malposition, instability, patellofemoral overstuffing, loosening or wear, or non-responsive stiffness with correctable CT findings [19,209,223]. Planning demands assessment of component position and rotation, ligament integrity, bone stock and patellar tracking, with extensile exposure where needed, aiming to restore the joint line, balance gaps and provide adequate constraint (commonly posterior-stabilized or constrained condylar designs) [31]. In carefully selected patients revision restores arcs to roughly 90–110° (gains about 28–50°), though pooled estimates are lower (Ghani 24.7°, versus 43.4° for open arthrolysis) because revision cases are more complex [3,9,19,29,32]. Complication rates exceed those of primary surgery, and residual pain and dissatisfaction are not uncommon even when ROM improves, with arthrodesis rarely required [18,32]. Crucially, a subset with stiff, painful TKAs have pain disproportionate to mechanics, widespread hyperalgesia, neuropathic features and strong psychosocial drivers; revising these knees without a clear mechanical target risks persistent pain, so pain phenotyping and psychological assessment must precede the decision, and without correctable mechanics, multidisciplinary pain management is more appropriate than further surgery [18,216,221]. Revision for stiffness should therefore be reserved for demonstrable mechanical pathology and realistic expectations, within an integrated strategy addressing rehabilitation, pain modulation and psychosocial factors.

## 11. Management of Chronic Pain After TKA

Management of chronic pain after total knee arthroplasty (TKA) should begin only after infection, fracture, loosening, gross instability, extensor failure and correctable mechanical or fibrotic causes have been considered. This does not make chronic postsurgical pain (CPSP) a diagnosis of exclusion or imply that pain without an obvious implant problem is “non-organic”; persistent post-TKA pain is often a mixed biopsychosocial condition combining residual nociceptive input, neuropathic pain, central sensitization, sleep disturbance, psychological distress, deconditioning and social context [16,23,41,42,58,109]. The goal is to reduce pain interference and restore function rather than eliminate all pain, with treatment phenotype-guided, since a focal neuroma, a CRPS-like presentation, widespread nociplastic pain and low-grade synovitis demand different pathways [16,23,51,105,107,108,109].

### 11.1. Multidisciplinary and Pathway-Based Care

Once a major surgically correctable cause is excluded or treated, chronic post-TKA pain should be managed through a coordinated pathway rather than fragmented consultations, with the surgeon remaining engaged—patients should not be abandoned because revision is not indicated. The STAR pathway is the most relevant structured model: it identifies troublesome post-replacement pain, performs systematic assessment and delivers treatment matched to likely mechanisms and functional barriers, with a favorable cost and feasibility signal [58,61,82]. Practical assessment covers the index operation and imaging, infection and mechanical re-exclusion, pain mapping and neuropathic screening, other painful sites, sleep, mood, catastrophizing, fear of movement and opioids, and functional testing—yielding a dominant phenotype: nociceptive/mechanical, neuropathic, CRPS-like, centrally sensitized or mixed [16,23,41,42,51,58].

### 11.2. Pharmacologic Management

Medication should support rehabilitation, sleep and function rather than replace diagnostic reasoning. For persistent nociceptive pain—particularly with synovitis, tendinopathy or inflammatory flares—acetaminophen, NSAIDs or COX-2 inhibitors may be used when safe, mindful of renal, cardiovascular and gastrointestinal risk [109,173,179,180]. Neuropathic features support topical lidocaine or capsaicin, tricyclic antidepressants, duloxetine and gabapentinoids in selected patients, titrated with review for sedation, falls and interactions [23,109,174]. Long-term opioids should generally be avoided or reviewed, since preoperative and prolonged postoperative use predict worse pain, poorer function and persistent use; need beyond healing should prompt reassessment and gradual tapering with non-opioid analgesia rather than abrupt withdrawal [151,152,153,154,155,156,157].

### 11.3. Psychological and Behavioral Interventions

Psychological care is not an admission that pain is imaginary; it targets the mechanisms that maintain pain and disability—catastrophizing, fear of movement, hypervigilance, low self-efficacy, depression, anxiety, insomnia and avoidance—consistently linked to worse outcomes and reduced rehabilitation engagement [2,14,16,41,42,55]. Cognitive-behavioral and acceptance-based approaches help patients reframe pain, manage flares, improve pacing and reconnect with valued activities, and multidisciplinary programs incorporating them benefit selected patients despite limited TKA-specific evidence [58,61,224]. As in Section 9, pain neuroscience education works best with exercise and graded exposure, and sleep warrants explicit attention (hygiene, CBT for insomnia, treatment of apnea), since maladaptive sleep–pain behaviors predict later pain [42,111,130,145,225,226].

### 11.4. Exercise, Graded Activity and Long-Term Rehabilitation

Exercise remains central but must be matched to pain phenotype, since generic strengthening may be insufficient with widespread pain, fear of movement, insomnia, neuropathic pain or pain-limited stiffness; a comprehensive program spans quadriceps and hip strengthening, aerobic conditioning, balance, gait, flexibility, graded exposure and pacing [111,130,224]. For centrally sensitized or widespread-pain phenotypes, the first goal is activity consistency rather than high-load strengthening, boom–bust patients setting a non-provocative baseline and progressing on a planned schedule rather than day-to-day pain [61,111,224]. For pain-limited stiffness, ROM work should combine analgesic timing, desensitization and graded exposure, since forceful stretching in a fearful patient reinforces guarding while avoidance permits secondary contracture [28,41,42,52]. Long-term care may need booster physiotherapy rather than indefinite unsupervised exercise [58,61,192,193,224].

### 11.5. Interventional and Neuromodulatory Procedures

Interventional procedures may help selected patients but should not substitute for diagnosis, and suits cases where infection, loosening, major instability and revision-worthy failure are excluded and the suspected generator matches the intervention. Diagnostic injections are informative: local anesthetic around a focal neuroma, tendon, bursa or genicular nerve distinguishes peripheral generators from diffuse nociplastic pain, a strong but temporary response guiding treatment [105,227,228]. Genicular nerve radiofrequency ablation (RFA) has been used for chronic knee pain including selected painful TKA cases, but TKA-specific evidence is limited and selection critical, with caution in CRPS-like or poorly defined widespread pain [23,229,230,231]. Available post-TKA evidence includes conventional thermal and cooled genicular radiofrequency ablation; whereas, pulsed radiofrequency has a weaker and less directly applicable evidence base; in the multicenter COCOGEN randomized trial including patients with knee osteoarthritis or persistent postsurgical pain after TKA, conventional and cooled radiofrequency produced broadly similar overall responder rates at 12 months, although a signal favoring cooled radiofrequency was reported in the persistent postsurgical pain subgroup, and these findings remain hypothesis-generating and do not establish superiority of one modality for post-TKA pain [232]. Geniculate artery embolization for synovial hypervascularity shows preliminary promise [233], and spinal cord or dorsal root ganglion stimulation is reserved for highly selected refractory neuropathic or CRPS-like cases after a multidisciplinary pathway has failed [23].

### 11.6. Special Scenarios

#### 11.6.1. Neuroma-Related Pain and Pseudo-Stiffness

Neuroma-related pain, especially of the infrapatellar branch of the saphenous nerve, can masquerade as stiffness: patients avoid flexion, kneeling or touch because movement stretches a painful cutaneous nerve or triggers allodynia, while passive joint motion remains available [104,105]. The pattern is focal incisional or anteromedial pain, numbness, burning, electric shocks, allodynia and a positive Tinel sign, and diagnostic nerve block is central—substantial temporary relief supports a peripheral generator and may predict benefit from topical therapy, desensitization, neuropathic medication, image-guided blocks, neuroma excision, relocation or selective denervation [105,227,228]. The key pitfall is treating neuroma-driven pseudo-stiffness as arthrofibrosis, since forceful MUA, arthrolysis or revision may worsen neuropathic pain when the problem is nerve injury rather than mechanical block.

#### 11.6.2. Complex Regional Pain Syndrome

CRPS after TKA is uncommon but potentially devastating and should be suspected when pain is disproportionate with allodynia, hyperalgesia, swelling, color or temperature asymmetry, sweating change, motor dysfunction or trophic features [23,109,110]. ROM may be profoundly limited, but the limitation is often pain-limited and autonomic rather than fixed arthrofibrosis. Management should be early and multidisciplinary—education, edema control, desensitization, graded loading, graded motor imagery or mirror therapy, neuropathic medication, sleep and mood treatment and specialist referral, with sympathetic blocks or neuromodulation if refractory [23,109,234,235]. Aggressive manipulation or revision in unrecognized CRPS can worsen symptoms, so CRPS should be screened before forceful stiffness interventions.

#### 11.6.3. Chronic Pain After Multiple Revisions: When to Stop Operating

Patients with persistent pain after multiple revisions are among the hardest to manage, with scarring, extensor compromise, altered anatomy, opioid exposure, mood disturbance, widespread pain and lost confidence; further surgery may be technically possible but clinically unhelpful. Before another revision, the case should be reviewed in a multidisciplinary setting, reassessing infection, loosening, instability, malrotation, extensor failure, neuroma, referred pain and CRPS. If no clear mechanical, infective or fibrotic target is found and the dominant phenotype is central, neuropathic or psychosocial, the clinician should state plainly that further revision has a low probability of meaningful relief and a real risk of worsening pain, stiffness, infection or disability [23,35,82,109,224]. Stopping surgery should not mean abandoning the patient: it means redirecting care toward pain rehabilitation, medication rationalization, sleep and mood treatment, graded activity and realistic goals—a difficult but ethically necessary conversation that protects against iatrogenic harm while preserving the therapeutic alliance.

## 12. Future Directions, Implementation, Strengths and Limitations

Chronic pain and stiffness after TKA are a convergent endpoint of biological, mechanical and psychosocial processes that remain only partly understood: definitions are inconsistent, well-described risk factors are rarely integrated into usable prediction tools, the biology of fibrosis and central pain is incompletely mapped, and promising antifibrotic, digital, psychological and interventional strategies have mostly been tested in small or uncontrolled series [14,35,52,91,194,224]. Key research priorities are summarized in Table 7 and elaborated below.

First, consensus definitions and core outcome sets are needed, since stiffness, arthrofibrosis and CPSP are applied inconsistently across ROM thresholds, time points and pain cut-offs; an OMERACT-like process should distinguish pain-limited, mechanically stiff and truly arthrofibrotic knees and agree patient-co-developed core outcomes for registries [14,35,38,52,194].

Second, risk prediction should move beyond single factors to validated multivariable models combining demographic, clinical, psychological, central-sensitization and mechanical variables with dynamic postoperative updating and stratified-care triage; externally validated, transparently reported machine-learning approaches integrating such multimodal, longitudinal data may improve individualized prediction over conventional scores [14,58,91,112,194,224,236].

Third, biomarkers and advanced imaging are required to phenotype fibrosis and pain, since none currently distinguishes early arthrofibrosis from expected scarring or predicts non-operative response; priorities include profibrotic markers (TGF-β1, connective-tissue growth factor, collagen fragments), standardized QST and validated MRI, ultrasound and dynamic-fluoroscopy phenotypes [35,52,91,112,237].

Fourth, novel pharmacologic and biologic antifibrotic therapies could, in principle, break the cycle of repeated mechanical procedures for true fibrotic disease, but the evidence remains preliminary. Candidate approaches—collagenase (by analogy with Dupuytren’s contracture), relaxin-2, and agents targeting TGF-β, connective-tissue growth factor, IL-6 or TNF-α—rest on biological plausibility rather than proven efficacy: to the best of our knowledge, no completed Phase 2 or 3 randomized controlled trial has established any antifibrotic agent for post-TKA arthrofibrosis, and the Dupuytren analogy is imperfect because intra-articular scar biology and joint biomechanics differ. As noted in Section 9, the human evidence is indirect—perioperative anti-inflammatory agents are associated observationally with lower MUA or lysis rates; whereas, a randomized trial after MUA did not improve range of motion—while a dedicated randomized trial of losartan after TKA (NCT06108063) is ongoing. Progress requires preclinical models replicating implant–tissue biomechanics and carefully designed early-phase trials with rigorous safety monitoring [35,91].

Fifth, digital health and telerehabilitation can supply infrastructure for continuous phenotyping: platforms collecting daily pain, activity and ROM (e.g., Lebleu’s 740-patient cohort across three countries) identify at-risk trajectories early, and telerehabilitation matches conventional physiotherapy for short-term ROM and function. Crucially, detecting an at-risk trajectory is not the same as improving outcome—randomized evidence that digital monitoring itself changes chronic pain, stiffness or HRQoL is limited—so these tools should trigger defined clinical action rather than passively collect data, and risk-stratified digital pathways need testing against in-person care for outcomes, cost and equity [194,238,239,240].

Sixth, large stratified trials of psychosocial and educational interventions are needed, since the biopsychosocial model has strong observational but modest interventional support: a combined exercise-plus-education trial was no better than education alone, and an uncontrolled multidisciplinary program called explicitly for randomized testing [41,111,224]. Adequately powered trials of pain neuroscience education, cognitive-behavioral and acceptance-based therapy and combined exercise–psychology programs are required, with attention to moderators, delivery and economics [14,41,111,224].

Seventh, implementation science must embed effective pathways into routine care rather than specialist clinics. The STAR pathway—three-month questionnaire screening plus structured telephone assessment and coordinated referral—was cost-effective and scalable, yet referral often remains ad hoc rather than triggered by risk scores [58,194,224]. Realistic implementation must address concrete barriers: staffing and funding for screening and coordination, integration with referral routes, reliable three-month case-finding, and clear pathway ownership; strategies should be tested with cluster-randomized or stepped-wedge designs, using registries to build a learning healthcare system [14,58,194,224,241].

### Strengths and Limitations of This Review

Beyond these research priorities, two surgical-technique questions warrant balanced study. Robotic and computer-assisted systems improve alignment, rotation and gap-balancing accuracy and may reduce malrotation- or imbalance-related symptoms, but current randomized evidence shows better alignment without a clear advantage in chronic pain, function or satisfaction over conventional technique; and routine versus selective patellar resurfacing should be individualized to anterior-knee-pain risk, patellofemoral morphology and revision risk rather than applied uniformly.

The principal strength and central novel contribution of this review is the integration of chronic pain and stiffness after TKA into a single, clinically oriented phenotypic framework. These problems have been characterized almost entirely in isolation, with earlier classifications addressing only narrow facets such as the timing of dissatisfaction or the severity of fixed flexion deformity. To our knowledge no previous review has combined the six phenotypes proposed here into a unified schema linking each phenotype to its dominant mechanism, key discriminators and a management direction (Table 4; Figure 3). By operationalizing the biopsychosocial model into a bedside-applicable structure, the framework foregrounds the distinctions that most determine outcome—pain-limited versus mechanically restricted motion, and structurally correctable versus centrally driven pain—and offers a shared vocabulary and testable structure for prospective validation.

This review also links mechanisms to practical diagnostic and management pathways, emphasizing clinically important distinctions such as pain-limited versus true mechanical stiffness, neuropathic versus nociplastic pain, and revision-worthy mechanical failure versus structurally acceptable but painful TKA.

Several limitations should be acknowledged. This is a structured comprehensive narrative review and thematic synthesis, not a systematic review or meta-analysis: the search was broad but not preregistered, article selection was not performed through dual independent screening, and no standardized risk-of-bias tool was applied to every study, so non-English, unpublished and negative or inconclusive reports may be under-represented. The literature is heterogeneous, with inconsistent definitions, variable follow-up and diverse outcomes [8,9,12,13,14,34,35,38,52], and many studies are observational, retrospective or single-center, with randomized trials still limited for psychological interventions, chronic pain pathways, digital monitoring, antifibrotic therapies and stiffness-specific rehabilitation. Finally, the proposed phenotypes and algorithms are evidence-informed but not prospectively validated; they are intended to structure clinical reasoning and research design, not to replace multidisciplinary judgment or local guidelines.

## 13. Conclusions

Chronic pain and stiffness after total knee arthroplasty are clinically important “hidden failures” not adequately captured by implant survivorship, radiographic fixation or revision rates alone. Although TKA relieves pain and restores function for most patients, a meaningful minority experience persistent pain, stiffness, dissatisfaction and reduced quality of life despite apparently successful surgery: clinically meaningful pain affects roughly 10–30% of recipients; whereas, stiffness and arthrofibrosis are less frequent (about 1–7% depending on definition) but disproportionately drive rehabilitation burden, reintervention, readmission and cost. These symptoms are seldom explained by one cause; they arise from interacting mechanical, fibrotic, infective, neuropathic, central and psychosocial mechanisms rather than a binary distinction between “failed implant” and “unexplained pain”.

The central message is a shift from a narrow implant-centered model toward an integrated, phenotype-based framework. Because mechanical, fibrotic, neuropathic, central and psychosocial contributors frequently overlap in the same knee, a practical approach begins by phenotyping into the dominant modifiable driver (Section 7), recognizing that many patients occupy more than one domain; the aim is not a rigid label but clarity about which mechanisms are dominant and modifiable, and where further surgery is more likely to harm than help. Prevention must span the whole perioperative pathway; diagnosis must move beyond “painful” or “stiff” to a phenotype-oriented work-up that excludes infection and mechanical failure while actively screening for neuropathic, central and psychosocial contributors; and management should be stepwise and matched to phenotype—intensified rehabilitation, bracing and manipulation for evolving fibrotic stiffness; arthrolysis for mature arthrofibrosis with sound components; revision only for a clear mechanical target; and multidisciplinary pain pathways rather than revision when the dominant phenotype is centrally sensitized, neuropathic or psychosocial.

More broadly, surgeons, physiotherapists, anesthetists, pain physicians, psychologists and primary-care clinicians need a shared language for chronic pain and stiffness after TKA. “No revision indicated” must not mean “no further care needed”; patients with persistent symptoms need legitimate pathways that validate their experience, identify modifiable drivers and redirect care toward appropriate mechanical, rehabilitative or pain-focused strategies, as structured models such as the STAR pathway demonstrate. Future progress will depend on consensus definitions, core outcome sets, prospective phenotype validation, risk-prediction tools, fibrosis biomarkers and pragmatic implementation trials—supporting a learning, patient-centered arthroplasty system in which high-risk patients are identified early, symptoms are phenotyped, mechanical and infective problems are corrected when present, pain and rehabilitation pathways are activated when surgery is unlikely to help, and outcomes are tracked beyond revision status: the best opportunity to reduce unnecessary reoperations, improve satisfaction and help more patients achieve durable pain relief, functional mobility and meaningful participation in life.

## Figures and Tables

**Table 1:** Working definitions and functional thresholds used in this review.

Term	Working Definition	Practical Threshold or Clue	Clinical İmplication
Typical TKA recovery	Expected improvement in pain, ROM and function without major complication	Major pain reduction by 3 months; functional ROM by 3–6 months	Deviation should trigger structured reassessment
CPSP after TKA	Knee-related pain persisting or emerging ≥ 3 months after surgery, not better explained by another cause	VAS/NRS ≥ 3/10, ≥30/100, or meaningful pain interference	Requires pain-mechanism and mechanical phenotyping
Stiffness after TKA	Function-limiting ROM restriction, regardless of cause	Flexion < 90–100° and/or fixed flexion contracture >10–15°	Determine mechanical, fibrotic or pain-limited pattern
Acquired idiopathic stiffness (AIS)	Function-limiting ROM loss persisting after exclusion of infection, component malposition, instability, loosening, heterotopic ossification and major extra-articular pathology; an exclusion-based clinical category rather than a tissue diagnosis	Flexion < 90–100° and/or FFC > 10–15° with no identifiable structural or infective cause after structured work-up	A broader clinical umbrella that may include true arthrofibrosis but is not synonymous with it; maps mainly to stiffness-dominant and arthrofibrosis-with-true-mechanical-block phenotypes, and to the combined stiff-and-painful phenotype when pain is prominent
Arthrofibrosis	Pathological fibrotic scar response causing persistent ROM loss	Firm end-feel, limited passive ROM, failure of standard rehabilitation	May require MUA, arthrolysis or revision if established
Pain-limited stiffness	Apparent ROM loss, driven mainly by pain, fear, guarding or inhibition	Passive ROM > active ROM; soft end-feel; improves with analgesia/relaxation	Prioritize pain control, graded exposure and rehabilitation
True mechanical stiffness	Fixed structural limitation of passive ROM	Firm end-feel; restriction persists under anesthesia; fibrosis/HO/malposition	Mechanical intervention more likely required
Functional ROM	ROM sufficient for daily activities	~65° gait; ~90° basic ADLs; 100–110° chair/stairs; >120° high-demand	ROM targets should be patient- and activity-specific

**Table 2:** Epidemiologic estimates and clinical/economic burden of chronic pain and stiffness after TKA.

Outcome Domain	Approximate Frequency	Main Clinical Consequence	Main Resource Consequence
Any persistent pain	30–50% in some cohorts	Ongoing symptoms, activity limitation, reduced satisfaction	Repeated consultations, analgesics, investigations
Moderate-to-severe CPSP	~10–30%	Poor function, impaired sleep, reduced HRQoL, dissatisfaction	Pain care, rehabilitation, medication, interventional procedures
Neuropathic-like pain among painful TKAs	One third to one half of those with persistent pain	Burning pain, allodynia; poor response to purely mechanical treatment	Neuropathic medication, nerve blocks, pain-clinic referral
Clinically meaningful stiffness	~1–7%, by definition	Difficulty with stairs, chair rise, transfers, kneeling, gait	Physiotherapy, bracing, MUA, arthrolysis
Acquired idiopathic stiffness	~4%	Persistent ROM loss after exclusion of identifiable causes	High share of non-revision reoperations and readmissions
Early severe stiffness/ankylosis	≤0.3%/~0.1%	Severe disability, poor long-term ROM, inferior survivorship	Multiple interventions, revision, prolonged rehabilitation
Stiff-and-painful TKA	Lower frequency, high impact	Severe disability, dissatisfaction, complex revision decisions	High-cost pathway: imaging, MUA, arthrolysis, revision
Dissatisfaction after primary TKA	Contemporary ~7–14%; historical/uncertain-inclusive cohorts ~17–20%	Perceived failure despite implant survival	Repeat assessment, pain and rehabilitation services

**Table 3:** Key clinically actionable risk factors and early triggers for chronic pain and stiffness after TKA. Factors are grouped by clinical risk domain and perioperative phase for rapid clinical use; the complete risk-factor matrix, with separate gradings of association for chronic postsurgical pain and for stiffness, is provided as Supplementary Table S1. Abbreviations: BMI, body mass index; CBT, cognitive behavioral therapy; CPM, conditioned pain modulation; CT, computed tomography; FFC, fixed flexion contracture; MUA, manipulation under anesthesia; PJI, periprosthetic joint infection; PNE, pain neuroscience education; PPT, pressure pain threshold; PT, physiotherapy; ROM, range of motion; TKA, total knee arthroplasty.

Risk Domain	Key Factor or Trigger	Main Phenotype İmplication	Modifiable or Actionable?	Practical Response
Demographic	Younger age/high activity demand	Pain-dominant; unmet-expectation dissatisfaction	No (counsel)	Counsel on realistic activity goals and expectations
Demographic/metabolic	Female sex or raised BMI/metabolic risk	Stiffness- and pain-dominant; combined	Partly	Optimize weight and glycemia; recognize risk without stereotyping
Pain profile	High preoperative knee pain or pain at rest/night	Pain-dominant; centrally sensitized	Partly	Preoperative pain optimization; screen sleep, mood, central sensitization
Pain/sensory profile	Multiple painful body sites; low PPT, impaired CPM	Centrally sensitized; nociplastic and neuropathic overlap	Partly (research-level)	Identify widespread/nociplastic phenotype; specialist pain-risk stratification
Psychological/behavioral	Catastrophizing, anxiety–depression, kinesiophobia	Pain-dominant; centrally sensitized; amplifies all phenotypes	Yes	PNE, CBT-informed and psychologically informed rehabilitation, graded exposure
Sleep	Insomnia or maladaptive sleep–pain behavior	Centrally sensitized; pain-dominant	Yes	Sleep intervention; analgesia timing
Joint status	Severe preoperative flexion loss or FFC	Stiffness-dominant; arthrofibrosis with mechanical block	Partly	Prehabilitation; extension-focused planning; counsel residual stiffness risk
History/diagnosis	Prior surgery, trauma or infection; inflammatory or post-traumatic knee	Stiffness-dominant; arthrofibrosis; combined	Mostly no	Anticipate scarring; infection vigilance; complex planning, realistic ROM targets
Surgical	Component malrotation, patellofemoral overload or gap imbalance	Combined stiff-and-painful; mechanical driver	Yes	Precise technique and gap balancing; CT if symptomatic
Surgical/early postoperative	Hemarthrosis, effusion, wound problem or suspected PJI	Stiffness-dominant; arthrofibrosis; infection-driven	Partly	Hemostasis; prompt work-up and treatment of infection or effusion
Early postoperative	High acute postoperative pain	Pain-dominant; centrally sensitized	Yes	Rapid analgesic escalation; acute pain pathway
Early postoperative	Flexion < 90° at 4–6 weeks or early ROM plateau	Stiffness-dominant; early arthrofibrosis	Yes	Intensified PT, bracing; consider early MUA
Rehabilitation/system	Poor rehabilitation access/adherence or fear-driven under-use	Stiffness- and pain-dominant	Yes (system-level)	Structured follow-up, supervised PT, telerehab; graded activity and pacing

**Table 4:** Clinical phenotypes of the painful and/or stiff TKA. Abbreviations: ACT = acceptance and commitment therapy; CBT = cognitive behavioral therapy; CPM = conditioned pain modulation; CRPS = complex regional pain syndrome; FFC = fixed flexion contracture; HO = heterotopic ossification; IBSN = infrapatellar branch of the saphenous nerve; MUA = manipulation under anesthesia; PF = patellofemoral; ROM = range of motion.

Phenotype	ROM Profile	Pain Character and Distribution	Dominant Mechanism/Substrate	Key Discriminator	Primary Management Direction
Pain-dominant TKA with preserved ROM	Flexion ≥ 100–110°, FFC < 10–15°; no mechanical block	Localized anterior/medial/lateral or diffuse pain; often activity-related during stairs, chair rise or loaded flexion	PF overload/maltracking, internal rotation, synovitis, soft-tissue impingement, micro-instability ± early neuropathic/central features	Pain is dominant despite functional ROM; possible reproducible mechanical or soft-tissue target	Treat identified source; assess neuropathic/central overlay; avoid revision without a target
Stiffness-dominant TKA with modest pain	Flexion ~75–100° and/or FFC 10–20°; usually firm or mixed end-feel	Minimal rest pain; pain mainly at motion extremes	Residual capsular tightness, mild-to-moderate arthrofibrosis, extra-articular contracture, HO	Functional loss out of proportion to pain	High-intensity physiotherapy, static-progressive/dynamic bracing, selected MUA or arthrolysis
Combined stiff-and-painful TKA	Often flexion < 75–90° ± FFC > 10–20°	Severe pain at motion extremes, often rest/night pain; high dissatisfaction	Mechanical error, gap imbalance, malrotation, arthrofibrosis ± low-grade infection ± central sensitization	Both passive ROM loss and clinically important pain; high reoperation/resource burden	Exclude infection; correct mechanical/fibrotic drivers; combine surgery/rehab with pain and sleep optimization
Neuropathic-dominant TKA	Preserved, mildly reduced or guarding-limited ROM	Burning, electric shocks, allodynia, numbness, painful scar or infrapatellar distribution	IBSN injury/neuroma, neuropathic pain; CRPS at severe end	Sensory signs, focal Tinel, allodynia, response to nerve block; stiffness often pain-limited	Neuropathic medication, desensitization, graded PT, diagnostic/therapeutic nerve block, selected neuroma or CRPS care
Centrally sensitized/widespread pain phenotype	Variable; often preserved ROM or soft pain-limited stiffness	Diffuse or disproportionate pain; multiple pain sites; fatigue, poor sleep, mood symptoms	Nociplastic pain, central amplification, impaired CPM, widespread hyperalgesia, psychosocial amplification	Poor structure–symptom match; widespread pain and psychological/sleep burden	Multidisciplinary pain pathway, CBT/ACT-informed care, sleep/mood treatment, graded activity; avoid revision without target
Arthrofibrosis with true mechanical block and modest pain	Severe active and passive restriction; often flexion < 75° and/or FFC ≥ 20°; abrupt firm end-feel	Modest at rest; pain mainly at end range	Dense capsular fibrosis, adhesions, extensor scarring ± HO; mature mechanical block	Passive ROM remains restricted despite relaxation/anesthesia	Early MUA if immature; arthroscopic/open arthrolysis; revision with release if component-related

**Table 5:** Diagnostic tools by suspected phenotype or driver.

Suspected Driver	Key Clinical Clues	First-Line Assessment	Advanced or Selective Assessment	Diagnostic Pitfall
PJI/low-grade infection	Late pain or stiffness, warmth, recurrent effusion, night/rest pain, wound history, recurrent stiffness after MUA/arthrolysis	CRP, ESR, radiographs, aspiration if suspected	Synovial cell count/differential, culture, alpha-defensin/leukocyte esterase, multiple intraoperative cultures	Low-grade PJI may mimic idiopathic arthrofibrosis
Gross mechanical failure	Progressive pain, instability, deformity, fracture symptoms, extensor dysfunction	Weight-bearing AP/lateral/skyline radiographs; full-length alignment views	CT for component position; revision planning imaging	Acceptable radiographs do not exclude rotation or soft-tissue causes
Component malrotation/PF overload	Anterior pain on stairs/chair rise, maltracking, loaded-flexion pain, preserved or pain-limited ROM	Skyline radiograph, patellar tracking exam	CT for femoral/tibial rotation, tibial slope and component position	Patellar tilt on plain radiographs may underestimate rotational pathology
Gap imbalance/instability	Giving way, recurrent effusion, pain with transitions, mid-flexion symptoms	Varus–valgus and sagittal stability testing, radiographs	Stress radiographs, dynamic fluoroscopy, gait analysis in selected cases	Patients may describe instability as pain or weakness
Arthrofibrosis/true mechanical stiffness	Restricted active and passive ROM, firm end-feel, failure of rehabilitation, limited motion under anesthesia	Goniometry, end-feel, infection screen, radiographs	CT for mechanical contributors; MRI/US for capsular thickening/soft tissue; examination under anesthesia	Do not diagnose idiopathic arthrofibrosis before excluding PJI and malposition
Pain-limited stiffness	Active ROM much worse than passive, soft/inconsistent end-feel, fear, guarding, pain behavior	Active/passive ROM comparison, pain/fear assessment	Short trial of analgesia, reassurance and graded physiotherapy; examination under anesthesia if unclear	Forceful MUA may worsen pain if dominant driver is pain/CRPS
Neuropathic pain/neuroma	Burning, electric shocks, allodynia, numbness, focal incisional or anteromedial tenderness, Tinel sign	Sensory exam, DN4 or painDETECT	Ultrasound, diagnostic local anesthetic nerve block	Neuropathic pseudo-stiffness may be mistaken for arthrofibrosis
CRPS-like presentation	Disproportionate pain, allodynia, swelling, color/temperature asymmetry, sweating change, motor dysfunction	Clinical assessment using Budapest criteria	Pain specialist assessment; exclude infection and mechanical causes	Aggressive revision or MUA may worsen unrecognized CRPS
Central/nociplastic pain	Widespread pain, multiple pain sites, insomnia, fatigue, poor structure–symptom match	Pain map, painful-site count, PCS, mood/sleep tools	QST/CPM in specialist or research settings; wearable activity/sleep data	Revision without a structural target is unlikely to address dominant mechanism
Extra-articular constraint	Prior fracture/osteotomy, extensor scarring, hip/spine pathology, posture-dependent ROM loss	Hip/spine/limb exam, gait assessment, radiographs	CT/MRI/US for HO, tendon/fascial scarring, malunion	Intra-articular arthrolysis alone may fail if extra-articular tethering dominates

**Table 6:** Escalation ladder for established stiffness after TKA. PT = physiotherapy; SPS = static-progressive stretch; NMES = neuromuscular electrical stimulation; IASTM = instrument-assisted soft-tissue mobilization; MUA = manipulation under anesthesia; PJI = periprosthetic joint infection; CRPS = complex regional pain syndrome; PCL = posterior cruciate ligament; KSS = Knee Society Score.

Step	Indication and Timing	Typical ROM Gain/Outcome	Key Risks and Caveats
Intensified non-operative (high-intensity PT; SPS/dynamic bracing; IASTM; NMES; pharmacological adjuncts)	Foundation for all; first line once mechanical/infective causes excluded	SPS ≈ 10–25° (≈17° flexion, ≈7° extension; ≈22° in one series); ≥90% avoid major surgery in selected cohorts	Time-bounded, goal-directed; adherence-dependent; drugs supportive only
2. MUA	Flexion < 90° at 6–12 wk, plateau, firm end-feel; best within ~3 months	Total arc ≈ 30–47° (≈38°; 29° flexion, 6° extension); functional > 90° in most; ~6% failure	Avoid in PJI, malposition, instability, CRPS, high fracture risk; gradual controlled force; rare fracture/tendon avulsion; post-MUA radiographs
3. Arthroscopic arthrolysis	Failed MUA or maturing contracture; isolated flexion deficit, focal adhesions/PCL scar, well-positioned components	≈25–42°, 73–88% success	Less effective for global/extension stiffness; re-adhesion if rehab inadequate
4. Open arthrolysis ± limited revision (± extensile exposure)	Severe/longstanding stiffness, extra-articular contracture, soft-tissue-dominant; components well-fixed and aligned	≈18–38°, 41–83% success (e.g., 64° → 94°, KSS 34 → 77); isolated insert exchange poor	Higher risk: infection, extensor insufficiency, fracture; counsel 90–110° goal, pain may persist
5. Revision arthroplasty	Correctable mechanical error: malposition, instability, overstuffing, loosening/wear	Series 28–50° (pooled ≈25°); restores ≈90–110°	Highest morbidity; residual pain/dissatisfaction common; not for central/non-mechanical pain—phenotype first

**Table 7:** Priority research agenda for chronic pain and stiffness after TKA.

Research Domain	Current Gap	Priority Action	Desired Output
Definitions	Inconsistent CPSP, stiffness and arthrofibrosis terminology	Multistakeholder consensus process	Standard definitions for trials, registries and clinical pathways
Core outcomes	PROMs incompletely capture pain quality, stiffness, sleep and participation	Develop TKA-specific core outcome set	Comparable datasets across studies
Risk prediction	Isolated predictors, few validated tools	Combine clinical, psychological, QST and digital variables	Actionable preoperative and postoperative risk models
Arthrofibrosis biology	No validated fibrosis biomarker	Tissue, synovial fluid, serum, imaging and transcriptomic studies	Biomarker-informed fibrosis phenotypes
Pain mechanisms	Neuropathic and central pain under-recognized	Phenotype-specific studies using DN4, painDETECT, QST and sleep tools	Better matching of treatment to pain mechanism
Digital monitoring	Early adverse trajectories not routinely detected	App/wearable-based pain, ROM and activity monitoring linked to escalation	Early alerts and stratified care
Prevention trials	Generic interventions dilute effects	Enroll high-risk patients using risk stratification	Efficient preventive pathways
Management trials	MUA, bracing, arthrolysis, revision and pain pathways studied heterogeneously	Phenotype-based pragmatic trials	Evidence-based treatment sequencing
Implementation	Effective models not routinely embedded	STAR-like and stiffness-specific pathway implementation studies	Learning arthroplasty care systems

## Data Availability

No new data were created or analyzed in this study. Data sharing is not applicable to this article.

## Supplementary Materials

The following supporting information can be downloaded at: https://www.mdpi.com/article/10.3390/jcm15145557/s1, Table S1: Comprehensive risk-factor matrix for chronic pain and stiffness after total knee arthroplasty; Table S2: Reported outcomes of individual manipulation, arthrolysis and revision series for the stiff total knee arthroplasty.

## Abbreviations

TKA	total knee arthroplasty
CPSP	chronic postsurgical pain
ROM	range of motion
MUA	manipulation under anesthesia
PJI	periprosthetic joint infection
CRPS	complex regional pain syndrome
QST	quantitative sensory testing
CPM	conditioned pain modulation
PROM	patient-reported outcome measure
HRQoL	health-related quality of life
CT	computed tomography
MRI	magnetic resonance imaging
MARS-MRI	metal-artifact-reduction MRI
US	ultrasound
DN4	Douleur Neuropathique 4 questionnaire
PCS	Pain Catastrophizing Scale
CBT	cognitive-behavioral therapy
ACT	acceptance and commitment therapy
PNE	pain neuroscience education
RFA	radiofrequency ablation
NSAID	non-steroidal anti-inflammatory drug
TGF-β	transforming growth factor-β
COS	core outcome set
IBSN	infrapatellar branch of the saphenous nerve
STAR	Support and Treatment After joint Replacement
AIS	acquired idiopathic stiffness
HO	heterotopic ossification
FFC	fixed flexion contracture
PPT	pressure pain threshold
PF	patellofemoral
