# Supplementary material for: Chronic Pain and Stiffness After Total Knee Arthroplasty: A Comprehensive, Phenotype-Based Review of Mechanisms, Diagnosis and Management
Source: J Clin Med. 2026 Jul 15;15(14):5557. doi: 10.3390/jcm15145557 (PMC13413427; doi:10.3390/jcm15145557)
Supplement: Supplementary file 1 [file jcm-15-05557-s001.zip › jcm-4402776-supplementary.pdf]

Journal of Clinical Medicine

MDPI

Supplementary Material

Table S1. Comprehensive risk-factor matrix for chronic pain and stiffness after total knee arthroplasty. +++, strong and consistent association; ++, moderate association; +, weak, inconsistent or indirect association; —, not clearly associated. CPSP, chronic postsurgical pain; AIS, acquired idiopathic stiffness; CPM, conditioned pain modulation; FFC, fixed flexion contracture; PJI, periprosthetic joint infection; PPT, pressure pain threshold; ROM, range of motion.

Domain	Risk factor	CPSP relevance	Stiffness/AIS relevance	Modifiable?	Practical action
Demographic	Younger age / high activity demand	++	+	No	Counsel about expectations and activity goals
Demographic	Female sex	+	++	No	Recognize higher risk without stereotyping
Metabolic	BMI ≥30 kg/m²	+	++	Partly	Weight optimization, technical planning, rehab support
Comorbidity	Diabetes / metabolic disease	+	++	Partly	Glycemic optimization, wound vigilance
Pain profile	High preoperative knee pain	+++	+	Partly	Pain optimization, expectation setting
Pain profile	Pain at rest or night	++	+	Partly	Screen for sleep, mood and central sensitization
Pain profile	Multiple painful body sites	+++	+	Partly	Identify widespread/nociplastic phenotype
Sensory profile	Low PPT, temporal summation, impaired CPM	+++	+	Research-level / partly	Specialist pain-risk stratification
Psychological	Catastrophizing	+++	++	Yes	PNE, CBT-informed rehabilitation, graded exposure
Psychological	Depression / anxiety	++	+	Yes	Screen and treat; consider pain psychology
Behavioral	Fear of movement / kinesiophobia	++	++	Yes	Psychologically informed rehabilitation
Sleep	Insomnia / maladaptive sleep–pain behavior	++	+	Yes	Sleep intervention, analgesia timing
Expectations	Unrealistic or unmet expectations	++	+	Yes	Shared decision-making and goal alignment
Joint status	Preoperative flexion <90°	+	+++	Partly	Prehabilitation, counsel residual stiffness risk
Joint status	FFC >10–20°	+	+++	Partly	Extension-focused planning and rehabilitation
History	Prior surgery / trauma / infection	+	+++	Mostly no	Anticipate scarring; infection vigilance
Diagnosis	Post-traumatic, inflammatory or ankylosed knee	+	+++	Partly	Complex surgical planning, realistic ROM targets
Surgical	Component malrotation	++	++	Yes	Precise technique; CT if symptomatic
Surgical	Flexion–extension gap imbalance	+	+++	Yes	Balanced gaps; avoid tight flexion/extension

Surgical	Joint-line elevation / tibial slope error	+	++	Yes	Restore joint line and slope where possible
Surgical	Patellofemoral overstuffing / maltracking	++	+	Yes	Optimize rotation, thickness and tracking
Surgical	Hemarthrosis / excess soft-tissue trauma	+	++	Yes	Hemostasis, soft-tissue preservation
Early postoperative	High acute pain	+++	++	Yes	Rapid analgesic escalation; acute pain pathway
Early postoperative	Flexion <90° at 4–6 weeks	+	+++	Yes	Intensified PT, bracing, consider early MUA
Early postoperative	Wound problem / PJI / effusion	++	+++	Partly	Prompt diagnostic work-up and treatment
Rehabilitation/system	Poor physiotherapy access or adherence	++	+++	Yes/system-level	Structured follow-up, supervised PT, telerehab
Rehabilitation/behavior	Underuse or boom–bust overuse	++	++	Yes	Graded activity and pacing

Supplementary Table S2. Reported outcomes of individual manipulation, arthrolysis and revision series for the stiff total knee arthroplasty. These per-study figures are summarized in the main text (Section 10) as the pooled ranges shown; this table preserves the individual-series detail.

Study [ref.]	n (knees)	Mean ROM gain	Success / outcome	Notes
**Manipulation under anesthesia (MUA)**				
Gu et al. [132]	1,488*	~30–47° total arc	~6% failure	*Meta-analysis (22 studies)*
Ghani et al. [32]	—	38.4° total (29° flexion, 5.7° extension)	—	*Pooled review*
Namba & Inacio [208]	—	Higher final flexion if MUA <3 months	—	*Timing effect*
Hurn et al. [213]	227	—	~17% required further intervention	*Contemporary series*
**Arthroscopic arthrolysis**				
Bae et al. [217]	13	42°	77%	
Williams et al. [218]	10	~30°	88%	*With posterior cruciate release*
Diduch et al. [219]	40	26°	73%	*At 20 months*
Pooled [217–219]	—	~25–42°	73–88%	*Across series*
**Open arthrolysis**				
Mont et al. [216]	18	31° (64°→94°)	45% good, 22% excellent (11% poor, 22% fair)	*KSS 34→77*
Babis et al. [222]	7	~20°	No good/excellent results	*Isolated insert exchange*
Pooled [3,9,19,29,216]	—	~18–38°	41–83%	*Across series*
**Revision arthroplasty**				
Ries & Badalamente [29]	6	50°	100%	
Christensen et al. [19]	11	43°	73%	
Kim et al. [9]	56	28°	93%	
Haidukewych et al. [3]	16	33°	66%	
Pooled (Ghani [32])	—	24.7° (vs 43.4° for open arthrolysis)	—	*Revision cases more complex*

* Patients rather than knees (pooled meta-analysis). ROM, range of motion; KSS, Knee Society Score; MUA, manipulation under anesthesia. Reference numbers correspond to the main reference list. Success denotes the study-defined proportion achieving a satisfactory functional arc (typically ≥90–110°) or the authors' stated success criterion; ROM gain is the mean increase in arc of motion. Pooled rows give the across-series ranges reported in Section 10.
